# Association Between Estrogen Receptors and GATA3 in Bladder Cancer: A Systematic Review and Meta-Analysis of Their Clinicopathological Significance

**DOI:** 10.3389/fendo.2021.684140

**Published:** 2021-10-08

**Authors:** Carina Bernardo, Fátima L. Monteiro, Inês Direito, Francisco Amado, Vera Afreixo, Lúcio L. Santos, Luisa A. Helguero

**Affiliations:** ^1^ Experimental Pathology and Therapeutics Group, Portuguese Oncology Institute – Porto, Porto, Portugal; ^2^ Division of Oncology and Pathology, Department of Clinical Sciences, Lund University, Lund, Sweden; ^3^ Institute of Biomedicine – iBiMED, Department of Medical Sciences, University of Aveiro, Aveiro, Portugal; ^4^ Mass Spectrometry Group, Associated Laboratory for Green Chemistry (LAQV) of the Network of Chemistry and Technology (REQUIMTE), Department of Chemistry, University of Aveiro, Aveiro, Portugal; ^5^ Center for Research and Development in Mathematics and Applications –CIDMA, Department of Mathematics, University of Aveiro, Aveiro, Portugal; ^6^ Department of Surgical Oncology, Portuguese Oncology Institute - Porto, Porto, Portugal

**Keywords:** bladder cancer, estrogen receptors, GATA3, tumour markers, immunohistochemistry

## Abstract

**Background:**

Estrogen receptors alpha (ERα) and beta (ERβ) and the cooperating protein GATA-binding factor 3 (GATA3) have been implicated in bladder carcinogenesis and tumour progression. GATA3 and ER have been functionally linked in the establishment of luminal fate in breast tissue, but to date their relationship in bladder cancer has not been established. This information will be useful to advance diagnostic and prognostic markers.

**Aim:**

To determine the relationship between the expression of ERα, ERβ and GATA3 in bladder cancer, disclose their prognostic and diagnostic value and their association with clinicopathological characteristics.

**Methods:**

A comprehensive literature search in PubMed database was performed for all immunohistochemical studies of ERα, ERβ and/or GATA3 in bladder cancer patients. We selected eligible studies in accordance with the PRISMA guidelines and evaluated methodological quality and risk of bias based on quality criteria from the reporting recommendations for tumour MARKer (REMARK) prognostic studies. Risk of bias assessment was performed using Review Manager 5. R software was used for all statistical analysis, the packages used were meta and dmetar for the standard meta-analysis, and netmeta for the network meta-analysis.

**Results:**

Thirteen studies were eligible for ERα, 5 for ERβ and 58 for GATA3 meta-analysis. Low grade tumours showed significantly lower ERα expression. GATA3 was widely expressed in bladder tumours, especially urothelial carcinomas, with higher expression of GATA3 in low grade and low stage tumours. Data was insufficient to determine the prognostic value of either ERα or ERβ, but GATA3-positivity was associated with higher recurrence free survival. A negative correlation between ERα or ERβ positivity and GATA3 expression was disclosed. Additionally, several sources of heterogeneity were identified, which can be used to improve future studies.

**Conclusion:**

The clinicopathological value of ERα and ERβ was inconclusive due to low availability of studies using validated antibodies. Still, this meta-analysis supports GATA3 as good prognostic marker. On the contrary, ERα-positivity was associated to higher grade tumours; while ERα and ERβ were inversely correlated with GATA3 expression. Considering that it has previously been shown that bladder cancer cell lines have functional ERs, this suggests that ERα could be activated in less differentiated cells and independently of GATA3. Therefore, a comprehensive analysis of ERα and ERβ expression in BlaCa supported by complete patient clinical history is required for the identification of BlaCa subtypes and subgroups of patients expressing ERα, to investigate if they could benefit from treatment with hormonal therapy.

**Systematic Review Registration:**

Prospero, CRD42021226836.

## Introduction

Bladder cancer (BlaCa) arises and progresses along two distinct pathways with distinct behaviour and molecular profile (1–3). Low grade, non-muscle invasive cancers (NMIBC) account for 75% of the cases at diagnosis and are characterized by good prognosis. However, patients frequently develop local recurrences requiring lifelong cystoscopy surveillance, and around 25% of the cases will ultimately progress to invasive disease (4). In contrast, muscle invasive tumour (MIBC) progress rapidly and have a high propensity for metastasis with 5-year survival rate less than 15%, even after radical cystectomy and systemic treatment (5–7). Cisplatin based chemotherapy has been the standard of care for MIBC for the past three decades. Recently, immune check point inhibitors and erdafitinib, an FGFR antagonist, have been approved and show therapeutic benefit for a small group of patients (8, 9). Still, the relative lack of molecular biomarkers and targeted therapies for BlaCa diagnosis and treatment (10, 11), renders the pathological assessment currently used insufficient to predict disease progression and response to therapy (12).

BlaCa risk is mainly associated with cigarette smoking and gender (13). It is 3 to 4 times more frequent in men than in women, with the excess risk in males remaining even after adjustment for known risk factors (14). Gene expression studies identified intrinsic basal and luminal subtypes of BlaCa that closely resemble corresponding subtypes of breast cancer (BC) (15–17). Luminal BlaCa is characterized by high expression of PPARγ and active estrogen receptor (ER) signalling pathway including expression of FOXA1, GATA3 and TRIM-24 (17). GATA3 is a marker of luminal cell differentiation in the breast and bladder (18) and together with FOXA1 are important mediators of PPARγ signalling to drive luminal fate in BlaCa (19). GATA3 loss is associated with an invasive less differentiated phenotype (20) and is mutated in ~5% of sporadic and ~13% of familial BC (21–23). It is unclear if estrogens have any protective effect because women are more likely to be diagnosed with invasive disease and have less favourable outcomes after treatments (24). However, ER activation requires both GATA3 and FOXA1 (25). Disclosing the functional connection between GATA3 and ER expression in BlaCa may improve the current tools for patient management, namely their eligibility for endocrine therapy used to inhibit ER-mediated proliferation.

The two ERs (ERα and ERβ) are expressed in the normal urothelium of both sexes (26). Analysis of the TCGA urothelial cancer data set (n=406) showed that ERα and ERβ mRNA expression is low (median FPKM 0.2 and FPKM 0.1, respectively) but detected in about 80% of the samples. Moreover, several independent studies showed that, BlaCa-derived cells lines are responsive to anti-estrogenic therapy (27, 28). To date, few studies have assessed the association between ERα and ERβ protein with the clinicopathological features of BlaCa. The reports are inconsistent, and the role of ERs in BlaCa development and progression remains controversial, partly because many of the studies dealt with small and heterogeneous patient cohorts and used antibodies that were not validated for clinical diagnosis of ERα, or anti-ERβ antibodies that were proved to be unspecific at a later stage (29, 30).

A previous meta-analysis of immunohistochemical studies correlated ERβ expression with high grade (OR=2,169; p<0,001) and muscle-invasive (OR=3,104, p<0,001) tumours (31) and revealed associations between ERβ expression and worse recurrence-free (HR=1,573; p=0,013) and progression-free (HR=4,148; p=0,089) survivals in patients with NMIBC. However, these results are compromised due to inclusion of studies that used anti-ERβ antibodies that are unspecific (29). In the same study, incomplete information hampered conclusive evaluation of associations between ERα expression and patient’s clinicopathological features. Regarding GATA3, much effort has been devoted into understanding its prognostic value as immunohistochemical marker, but to date there is no systematic evaluation and meta-analysis of such findings. Additionally, there is no study assessing the relationship between these functionally related proteins. In this work, we present a systematic review of the literature and meta-analysis to investigate the associations between immunohistochemical detection of ERα, ERβ and GATA3 with clinicopathological features such as patient’s gender, age, tumour stage, grade and survival and explore the relationship between the expression of these three makers.

## Methods

This study was submitted to PROSPERO on January 7, 2021 and registered on February 7, 2021 (CRD42021226836).

### Search Strategy

The aim was to identify all primary literature that reported immunohistochemical detection of ERα, ERβ and GATA3 in BlaCa. All potentially relevant articles were identified by a search in PubMed/Medline database using both Medical Subject Headings (MeSH) terms and free text words in the search queries. Singular and plural forms of the key terms, searched in Title and Abstract, were combined with MeSH terms. For GATA3 the queries were (transitional cell carcinoma OR urothelial tumor OR urothelial cancer OR urothelial carcinoma OR bladder tumor OR bladder cancer OR bladder carcinoma OR urinary bladder neoplasms [MeSH Terms]) AND (GATA OR GATA3 OR GATA transcription factors [MeSH Terms]). For ERs, the queries combined all the MeSH Terms listed above for bladder cancer AND (receptors, estrogen OR estrogen OR estradiol OR oestrogen OR estrogen receptor ESR1 OR estrogen receptor beta ESR2 [MeSH Terms]). The search was unlimited for articles published up to December 2020. Existing reviews and reference lists were hand searched for studies missed by the initial query.

### Eligibility and Data Collection

**Figure 1:**
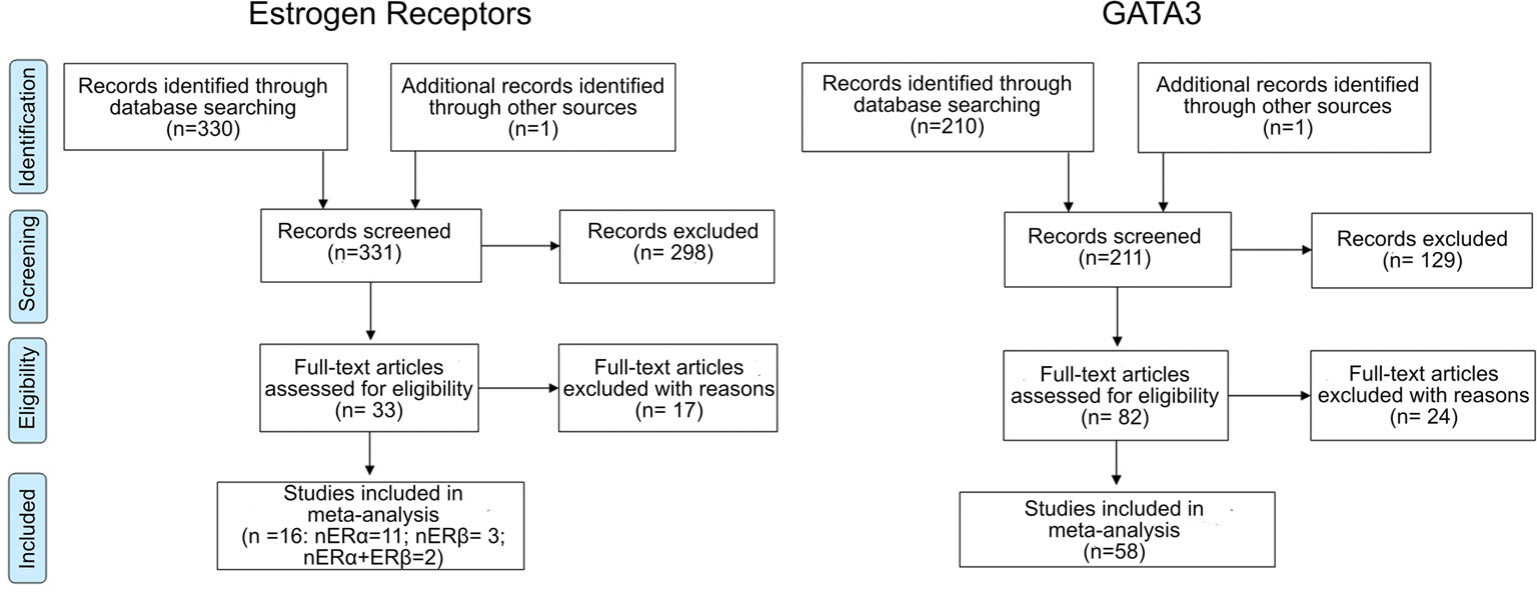
Flowchart summarizing the literature selection process for Estrogen Receptor (ERα and ERβ) and GATA-binding factor 3 (GATA3).

All retrieved references were screened for eligibility based on the title and abstract analysis by two of the authors. Potentially eligible full-text articles were retrieved for full-text assessment. The articles were reviewed against the following inclusion criteria: (1) expression level of ERα, ERβ or GATA3 analysed in human BlaCa samples by immunohistochemistry (IHC); (2) reports with sufficient data to evaluate the methodological quality of the trial and to carry out a meta-analysis, including a clear description of the study population and IHC methods (i.e. tissue handling, antibodies used, positive controls), and description of the methodology and cut-off used to assign expression status; (3) Correlation between ERα, ERβ and/or GATA3 expression and clinicopathological data discussed; (4) when different papers reported ERα, ERβ and GATA3 expression from the same patient cohort, the most recent or the most complete study was included. Only original reports were considered. Letters, reviews, case reports, editorials and comments were excluded. Selected references for which a full-text report was not available after contact with dedicated libraries and with corresponding authors were also excluded. For ERs, only published studies using validated antibodies were included. A flowchart depicting the literature search and selection process is represented in **Figure 1**.

For ERs, a total of 331 articles were identified, 298 were excluded after title and abstract screening for relevance. Of the 33 studies included in the qualitative analysis, 17 were excluded after full text analysis due to insufficient data, duplicated report of the same cohort, or use of non-validated or non-specific antibodies (**Table S1**). This resulted in 16 studies included of which 2 included information on ERα and ERβ (27, 32), 11 on ERα (33–41) and 3 on ERβ (42–44). For GATA3, 211 articles were retrieved, of which 129 were excluded after title and abstract screening. Of the 83 studies included in the qualitative analysis, 24 were excluded after full-text analysis due to insufficient data, duplicated report of same sample cohort, contradictory data between text and tables, and lack of information about antibody used (**Table S1**), resulting in 58 studies included. Three studies reported ERα and GATA3 in the same tumour sample cohort (35, 45, 46).

Data was extracted from all relevant articles independently by two authors using a predefined data collection template which included identification details (surname of first author, year of publication), number of cases (total number and number of positive cases), primary antibody and dilution used, cut-off for positivity, subcellular localization of the staining (cytoplasmic or nuclear), tissue used for analysis [whole section or tissue microarray (TMA)], tissue collection method [transurethral resection (TUR) and/or cystectomy (CYS)], expression levels according to clinicopathological features such as age, gender, tumour grade, stage, lymph node metastasis and histology. Tumour histology was grouped as pure urothelial carcinomas (UC), UC with divergent differentiation (UCDD) and variant histologies (VH) such as adenocarcinomas and pure squamous cell carcinomas. Prognostic data (duration of follow-up after surgery or treatment, endpoint, overall survival (OS), recurrence and progression-free survival) and the statistical analysis used in each study (type of statistical test, P-value, hazard or risk ratio, 95% confidence interval (CI), univariate or multivariate analysis) were also collected.

**Figure 2:**
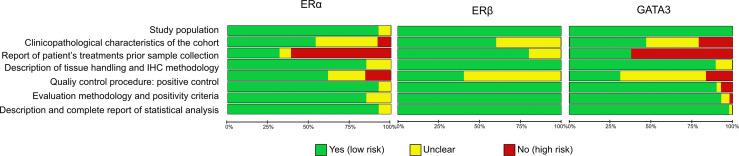
Risk bias assessment per item presented as percentages across all included studies.

The methodological quality and the risk of bias of each study were assessed independently by two of the authors using a list of quality criteria derived from the reporting recommendations for tumour MARKer (REMARK) prognostic studies and any disagreement was resolved by consensus. Four areas of potential bias were assessed: study design, assay methodology, results reporting and methods for statistical analysis. Risk of bias assessment was performed using Review Manager 5 (RevMan 5.3, Copenhagen: The Nordic Cochrane Centre, The Cochrane Collaboration, 2014). The overall risk of bias for an individual study was categorized as low (green: risk of bias low in all domains), unclear (yellow: risk of bias is unclear in at least one domain, but no domains with high risk) or high (red: high risk of bias in at least one domain) as shown in **Figure S1**. The weight of all studies on the overall risk of bias for each specific domain is shown in **Figure 2**.

### Data Analysis

All analysis were performed using R software (version 3.6.2) and the packages meta, dmetar (47) for statistics and netmeta for network meta-analysis (48). The prevalence, odds ratio (OR), Cohen’s d and relative risk (RR) were calculated as point estimates of the association between expression of ERα, ERβ or GATA3 and the patients’ clinicopathological characteristics. Pooled prevalence indicates the proportion of positive staining for each marker. Pooled OR was used to evaluate differences in the proportion of positive cases between pre-defined groups. Cohen’s d effect size was calculated relative to differences between the average age of the patients reported to be positive or negative where d = 0 means that distribution of ages in one group overlaps the distribution of ages in the other group. The effect size can further be interpreted as small (0.1), medium (0.5) and large (0.8), with higher values indicating less overlap between the groups (49). Pooled RR was calculated for differences in GATA3 positivity regarding Relapse-Free Survival (RFS). Between-studies heterogeneity was estimated using heterogeneity index (I^2^) statistics (50). In case of substantial heterogeneity between studies (I^2^>50%), only the results from random effects model were considered for further analysis; otherwise, a fixed effect model was used for the pooled statistical analysis and a meta-regression analysis (mixed-effects model) was performed using an ‘adjusted effect’ to potential moderators. All results were considered statistically significant at the level of 5% (p <0.05). Sensitivity analysis was carried out to assess the robustness of the results by removing individual studies from the meta-analysis and assessing the effect on the pooled results. The publication bias was evaluated using funnel plots and two-sided Egger’s tests (**Figure S2**).

Subgroup meta-analysis and/or meta-regression were performed to explore sources of heterogeneity using five factors: 1) antibody used, 2) cut-off for positivity, 3) tumour histology, 4) sample type and 5) sample collection. Meta-regression was also used to assess the influence of the following seven factors in the ERs and GATA3 proportion of positive cases: 1) gender, 2) tumour stage, 3) lymph node metastases, 4) tumour grade, 5) tumour histology, 6) therapy pre-collection, and 7) deaths by cancer.

To assess a possible relationship between the expression of ERα, ERβ and GATA3, we estimated OR using pairwise and network meta-analysis with random effects using frequentist methods. Moreover, we evaluated the inconsistencies between direct and indirect comparison using the z-value of test for disagreement (direct versus indirect) in network meta-analysis.

## Results

### Characteristics of the Eligible Studies for the Systematic Review

All studies were retrospective, 13 were eligible for ERα comprising a total of 1616 tumour samples (1998-2020; 20 - 317 patients per cohort), 5 for ERβ consisting of 675 samples (2006-2020; 80-224 patients) and 58 for GATA3 covering a total of 4254 samples (2011-2021; 4-303 patients), as shown in **Tables 1**–**3**, respectively.

**Table 1:** Characteristics of the studies included for meta-analysis of ERα expression in BlaCa.

Study	N	Positive Cases	Antibody	Collection	Sample	Cutoff criteria	Age (range)	Gender M/F	<T2/≥T2	LG/HG	Mets/no Mets	Histology UC/UCCD/VH	Treated no/yes	Country
Basakci (2002) (33)	121	15	K1900	TUR	tissue	10%	Med 62 (19-87)	99/22	121/0	112/9	NA	121/0/0	NA	NA
Bernardo (2020) (27)	80	14	6F11	CYS+TUR	tissue	1%	Mean 69.2 (38-86)	71/9	40/40	12/68	NA	80/0/0	67/13	Portugal
Bolenz (2009) (34)	198	9	1D5	CYS	tissue	NA		156/42	NA	14/184	63/135	198/0/0	138/60	NA
Borhan (2017) (35)	45	0	SP1	CYS+TUR	tissue	score	Mean 69.6 (51-83)	37/8	NA	NA	NA	0/0/45	NA	USA
Croft (2005) (36)	92	10	6F11	NA	tissue	10%	Mean 65 (30-93)	60/32	43/49	50/42	NA	92/0/0	92/0	USA
Imai (2019) (37)	125^A^	48	6F11	CYS+TUR	tissue	1%	(37-93)	89/26	81/44	63/62	NA	100/20/5	NA	Japan
Kaufmann (1998) (38)	185	34	6F11	NA	tissue	10%	Mean 68.3 (29-94)	84/101	138/47	140/45	NA	185/0/0	NA	Germany
Mashhadi (2014) (39)	120	3	1D5	CYS+TUR	tissue	10%	Mean 66.2 +- 12.1	105/15	61/59	20/100	14/106	120/0/0	120/0	Iran
Pena (2019) (46)	58^B^	14	SP1	TUR	TMA	1%	Mean 68 (47-89)	41/19	57/3	26/34	NA	60/0/0	NA	USA
Shen (2006) (32)	224	2	6F11	CYS+TUR	TMA	10%	NA	NA	145/79	114/96^C^	20/204	224/0/0	NA	NA
Tan (2015) (40)	317^B^	12	1D5	CYS	TMA	10%	Med 69 (37-90)	259/59	98/218^C^	28/262^C^	59/215^C^	314/0/4	242/76	USA
Wang Y (2020) (45)	31	3	1D5	NA	tissue	10%	NA	NA	NA	NA	NA	31/0/0	NA	USA
Wei (2009) (41)	20	0	6F11	NA	TMA	10%	NA	NA	NA	NA	NA	20/0/0	NA	TMA purchased from US Biomax (Rockville, MD)

M, male; F, female; <T2, non-muscle invasive tumours; ≥T2, muscle invasive tumours; LG, Low Grade; HG, High Grade; Mets, metastasis; UC, urothelial carcinoma; UCDD, urothelial carcinoma with divergent differentiation; VH, variant histology; NA, not available; TUR, transurethral resection of the bladder; CYS, cystectomy; TMA, tissue microarray; Med, median. ^A^Number of samples doesn’t correspond to number of patients; ^B^Not all samples were analysed for ERα; ^C^data not available for all samples, missing information for some samples.

**Table 2:** Characteristics of the studies included for meta-analysis of ERβ expression in BlaCa.

Study	N	Positive Cases	Antibody	Collection	Sample	Cutoff criteria	Age (range)	Gender M/F	<T2/≥T2	LG/HG	Mets/no Mets	Histology UC/ UCCD/ VH	Treated no/yes	Country
Bernardo (2020) (27)	80	73	14C8	CYS+TUR	tissue	1%	Mean 69.2 (38-86)	71/9	40/40	12/68	NA	80/0/0	67/13	Portugal
Izumi (2016) (42)	72	39	14C8	TUR	tissue	10%	Med 73 (63-80)	NA	72/0	50/18**^A^**	NA	72/0/0	36/36	Japan
Kontos (2011) (43)	111	84	14C8	CYS+TUR	tissue	10%	Mean 70 (23-90)	74/37	70/41	57/54	NA	111/0/0	111/0	NA
Miyamoto (2012) (44)	188	93	14C8	CYS+TUR	TMA	1%	Mean 65.9 (30-89)	148/40	97/91	56/132	32/53**^A^**	178/10/0	160/28	USA
Shen (2006) (32)	224	141	MYEB	CYS+TUR	TMA	10%	NA	NA	145/79	114/96**^B^**	20/204	224/0/0	NA	NA

M: male; F: female; <T2: non-muscle invasive tumours; ≥T2: muscle invasive tumours; LG: Low Grade; HG: High Grade; Mets: metastasis; UC: urothelial carcinoma; UCDD: urothelial carcinoma with divergent differentiation; VH: variant histology; NA: not available; TUR: transurethral resection of the bladder; CYS: cystectomy; TMA: tissue microarray; Med: median. **^A^** data not available for all samples, missing information for some samples. **^B^** data not available for all samples, missing information for some samples.

**Table 3:** Characteristics of the studies included for meta-analysis of GATA3 expression in BlaCa.

Study	N	Positive Cases	Antibody	Collection	Sample	Cutoff criteria	Age (range)	Gender M/F	<T2/≥T2	LG/HG	Mets/no Mets	HistologyUC/ UCCD/ VH	Treated no/yes	Country
Agarwal H. (2019) (51)	74	57	EPR16651	TUR	tissue	1%	Mean 55.9 (21-83)	65/9	NA	24/47**^C^**	NA	74/0/0	NA	India
Aphivatanasiri (2020) (52)	137	109	L50-823	NA	TMA	1%	Mean 70.5 (34-92)	101/36	NA	NA	NA	137/0/0	NA	Thailand, China and Indonesia*
Barth (2018) (53)	156**^A^**	151	CM405A	NA	TMA	10%	Med 70 (42–93)	104/28	156/0	NA	0/156	156/0/0	96/51**^C^**	Germany
Beltran (2014) (54)	20	20	L50-823	CYS+TUR	tissue	1%	Mean 63 (45-75)	14/6	0/20	NA	6/8**^C^**	0/20/0	NA	Spain, Portugal, Italy and USA*
Beltran (2014) (55)	28**^B^**	28	L50-823	CYS+TUR	tissue	1%	Mean 66 (45-83)	45/11	NA	NA	14/19	0/0/28	NA	Portugal, USA, Italy, Spain and France*
Bernardo (2019) (56)	205	191	D13C9	NA	TMA	10%	NA	156/49	163/40**^C^**	119/86	NA	194/10/1	NA	Portugal
Bertz (2020) (57)	33**^B^**	10	L50-823	CYS+TUR+Biopsy	tissue	NA	Mean 66.6 (24-88)	27/7	NA	NA	NA	0/16/18	NA	Germany
Bezerra (2014) (58)	22	7	L50-823	NA	tissue+ TMA	1%	Med 69.5 (34-88)	16/6	7/15	NA	4/18	0/22/0	NA	USA
Bontoux (2020) (59)	184**^A^**	94	L50-823	CYS	TMA	10%	Med 68 (40-86)	141/46	2/185	0/184 **^C^**	87/100	101/38/34 **^C^**	187/0	France
Borhan (2017) (35)	45	37	L50-823	CYS+TUR	tissue	Score (>1)	Mean 69.6 (﻿51-83)	37/8	NA	NA	NA	0/45/0	NA	USA
Broede (2016) (60)	25	21	L50-823	NA	TMA	Score (>2)	NA	NA	NA	NA	NA	16/0/9	NA	NA
Chang (2012) (61)	35	28	L50-823	NA	TMA	score	NA	NA	NA	0/35	NA	35/0/0	NA	NA
Clark (2014) (62)	27	23	L50-823	NA	TMA	score	NA	NA	NA	NA	NA	22/0/5	NA	TMA purchased from US Biomax (Rockville, MD)
Comperat (2017) (63)	32**^B^**	29	L50-823	CYS+TUR	tissue	10%	Mean 66.7 (38-84)	32/4	3/33	NA	7/17**^C^**	0/32/0	NA	France, Germany Czechia, USA and Canada
Davis (2016) (64)	79	56	L50-823	NA	TMA	1%	NA	NA	NA	NA	NA	79/0/0	NA	USA
Ellis (2013) (65)	49	12	L50-823	CYS	TMA	score	Mean 54 (30-79)	39/10	NA	NA	NA	0/0/49	NA	USA
Eckstein (2018) (66)	89**^B^**	46	L50-823	NA	TMA	score	Mean 69.7 (41-88)	69/26	0/95	0/95	58/29 **^C^**	41/52/2	68/27	Germany
Fatima (2014) (67)	22	16	L50-823	CYS	tissue	10%	NA	NA	NA	NA	NA	0/22/0	NA	USA
Guo (2020) (68)	74	52	HG3–31	NA	tissue	NA	NA	NA	NA	NA	NA	74/0/0	NA	USA
Gruver (2012) (69)	37	29	HG3-35	TUR	TMA	5%	NA	NA	NA	NA	NA	37/0/0	NA	USA
Gulmann (2013) (70)	50	22	HG3-31	TUR	tissue	5%	(34-96)	31/19	31/19	11/39	NA	15/23/12	NA	USA and Spain
﻿Gürbüz (2020) (71)	300	297	L50-823	TUR	tissue	20%	Mean 69 (28-100)	265/35	150/150	75/225	NA	300/0/0	300/0	Turkey
Hoang (2015) (72)	103	86	L50-823	NA	TMA	5%	NA	78/25	NA	26/77	NA	103/0/0	NA	USA
Jangir (2019) (73)	40	18	L50-823	CYS	tissue	20%	Mean 56.6	37/3	NA	0/40	17/23	22/18/0	40/0	NA
Johnson (2020) (74)	28	28	L50-823	CYS+TUR	tissue	1%	Med 66	24/3	1/16 **^C^**	NA	NA	0/0/28	4/23	USA
﻿Kandalaft (2016) (75)	21	21/20	L50-823/ HG3-31	NA	tissue	1%	NA	NA	NA	NA	NA	21/0/0	NA	USA
Kim (2020) (76)	166	92	L50-823	CYS+TUR	TMA	20%	Mean 76 (37-87)	139/27	0/166	7/159	NA	166/0/0	166/0	South Korea
Kim (2013) (77)	43	29	L50-823	TUR	TMA	5%	Mean 64.2 (52-79)	NA	NA	NA	NA	22/10/11	5/5**^C^**	South Korea
Leivo (2016) (78)	89	88	L50-823	CYS	TMA	5%	Mean 64 (43–85)	71/18	2/87	NA	43/46	89/0/0	56/33	USA
Liang (2014) (79)	244	114	HG3-31	CYS	TMA	10%	(32-90)	187/57	11/225**^C^**	NA	NA	103/141/0	NA	USA
Liu (2012) (80)	72	62	HG3-31	NA	TMA	5%	NA	NA	NA	NA	NA	72/0/0	NA	USA
Lobo (2020) (81)	70	62	HPA029731	CYS+TUR	tissue	10%	Mean 69.5 (45-91)	58/12	47/23	28/42	9/61	70/0/0	NA	Portugal
Lu (2020) (82)	176	176	UMAB218	CYS+TUR	tissue	score	Mean 62.1 (28-90)	153/23	176/0	40/136	7/169	100/76/0	33/143	China
Manach (2018) (83)	60	31	CM405B	CYS+TUR	TMA	10%	Mean 64.6 (41-91)	46/14	NA	NA	NA	32/28/0	54/6	France
Miettinen (2014) (84)	54	49	L50-823	NA	TMA	NA	NA	NA	NA	22/32	NA	49/5/0	NA	NA
Mitra (2018) (85)	5	5	390M-15	CYS+TUR	tissue	10%	Mean 66.8 (52-75)	5/0	NA	NA	NA	5/0/0	NA	NA
Miyamoto (2012) (86)	145	125	L50-823	CYS+TUR	TMA	1%	Mean 66 (30-89)	110/35	80/65	51/94	21/47**^C^**	145/0/0	128/17	USA
Mohammed (2016) (87)	79	56	L50-823	NA	TMA	20%	NA	NA	0/79	0/79	NA	79/0/0	NA	USA
Mohanty (2014) (88)	16	16	HG3-31	TUR	tissue	score	Mean 74.5 (45-79)	NA	0/16	0/16	NA	16/0/0	16/0	USA
Paner (2014) (89)	7	6	HG3-31	CYS	tissue	1%	Mean 67 (47-87)	6/1	0/7	NA	3/4	0/7/0	5/2	USA and Spain
Paner (2014) (90)	111	67	HG3-31	NA	TMA	5%	NA	NA	NA	NA	NA	10/20/81	NA	USA, Spain and South Korea
Patriarca (2014) (91)	11	11	L50-823	TUR	tissue	10%	Mean 74 (61-86)	7/4	11/0	10/1	NA	11/0/0	7/4	Italy and France
Rodriguez Pena (2019) (46)	58**^B^**	58	CM405B	TUR	TMA	1%	Mean 68 (47-89)	41/19	57/3	26/34	NA	60/0/0	NA	USA
Perrino (2019) (92)	26**^B^**	25	L50-823	CYS+TUR	tissue	1%	Med 68 (36-91)	56/13	1/68	NA	14/36 **^C^**	0/69/0	44/25	USA
Priore (2018) (93)	15	14	L50-823	NA	tissue	5%	Mean 72 (55-84)	15/1	10/5	9/6	NA	0/0/15	NA	USA
Rao (2013) (94)	36	3	L50-823	NA	tissue	1%	NA	NA	NA	NA	NA	0/0/36	NA	NA
Raspollini (2011) (95)	4	4	HG3-31	CYS+TUR	tissue	score	Mean 68.5 (53-78)	3/1	0/4	NA	2/2	0/0/4	2/2	NA
Samaratunga (2015) (96)	10	9	L50-823	TUR	tissue	score	NA	6/4	5/5	NA	NA	0/0/10	NA	Australia
Sanfrancesco (2016) (97)	26	16	L50-823	CYS+TUR	TMA	score	NA	NA	NA	NA	NA	0/0/26	NA	USA
Sjodahl (2017) (98)	303	194	D13C9	TUR	TMA	10%	NA	236/28 **^C^**	56/241**^C^**	41/262	NA	257/5/41	NA	Sweden
So (2013) (99)	12	10	L50-823	NA	tissue	score	Med 60.5 (26-85)	NA	NA	NA	NA	0/0/12	NA	USA
Verduin (2016) (100)	86**^A^**	43	L50-823	NA	TMA	1%	Mean 66.7 (39-91)	53/25	NA	NA	NA	0/17/69	NA	NA
Wang (2019) (101)	91	80	L50-823	CYS+TUR	tissue	10%	Mean 66 (39–89)	64/27	0/91	NA	31/60	91/0/0	91/0	Taiwan
Wang (2018) (102)	30**^B^**	1	HG3-31	CYS+TUR	TMA	NA	Mean 68 (34-90)	69/12	2/46**^C^**	42/39	27/54	0/0/30	NA	USA
Wang (2020) (45)	31	31	L50-823	NA	tissue	10%	NA	NA	NA	NA	NA	31/0/0	NA	USA
Yuk (2019) (103)	100	92	156-3C11	CYS+TUR	TMA	1%	Mean 65.1	83/17	0/100	NA	20/80	100/0/0	90/10	South Korea
Zhao (2013) (104)	69	62	HG3-31	NA	TMA	5%	Mean 68.7 (25-89)	45/24	NA	NA	69/0	48/18/3	NA	USA
Zinnall (2018) (105)	94	79	L50-823	NA	TMA	1%	Med 68 (41-99)	61/14**^C^**	7/74**^C^**	0/94	NA	0/0/94	NA	Germany

M: male; F: female; <T2: non-muscle invasive tumours; ≥T2: muscle invasive tumours; LG: Low Grade; HG: High Grade; Mets: metastasis; UC: urothelial carcinoma; UCDD: urothelial carcinoma with divergent differentiation; VH: variant histology; TUR: transurethral resection of the bladder; CYS: cystectomy; TMA: tissue microarray; Med: median. NA: not available. **^A^** Number of samples doesn’t correspond to number of patients; **^B^** Not all samples were analysed for GATA3; **^C^** data not available for all samples, missing information for some samples. *patients are from participating institutions but is doesn’t specify if all or just few and which ones.

### Methodological Quality and Risk of Bias

REMARK (106) based risk of bias assessment is shown **Figure 2** and **Figure S1**. The most common factor in the bias analysis was lack of information on pre-operative treatment status (high risk for ERα and GATA3 in over 50% of studies). Followed by positive controls (unclear in 25% of ERα studies and in over 50% of ERβ or GATA3 studies), incomplete description of clinicopathological characteristics of the cohort (specially for GATA3, with nearly 50% studies unclear or not reporting) and no information about the quality controls including positivity criteria (above 50% of ERβ or GATA3). The main differences in the methodology between studies included: use of different antibodies and antibody dilutions or different scoring systems.

### Meta-Analysis of ERα Expression in BlaCa

The pooled proportion of ERα-positive cases was 7%, (0-38%; **Figure S3**). Despite the high level of variation between the 13 studies (I2 = 93%), the sensitivity analysis did not identify any study as having a significant influence in the overall heterogeneity (**Figure S4A**). However, it is worth mentioning that data from Imai (2019) stood out and influenced pooled results, most likely due to the use of a lower cut-off (1%) and inclusion of UCDD tumours. Subgroup analysis could not explain the heterogeneity between studies (**Table S2**); Meta-regression disclosed lymph node metastases as a significant source of variation associated with ERα expression (p-value = 0.0275; mixed-effects model; **Table S3**).

#### Correlation of ERα Immunostaining With Clinicopathological Parameters

**Figure 3:**
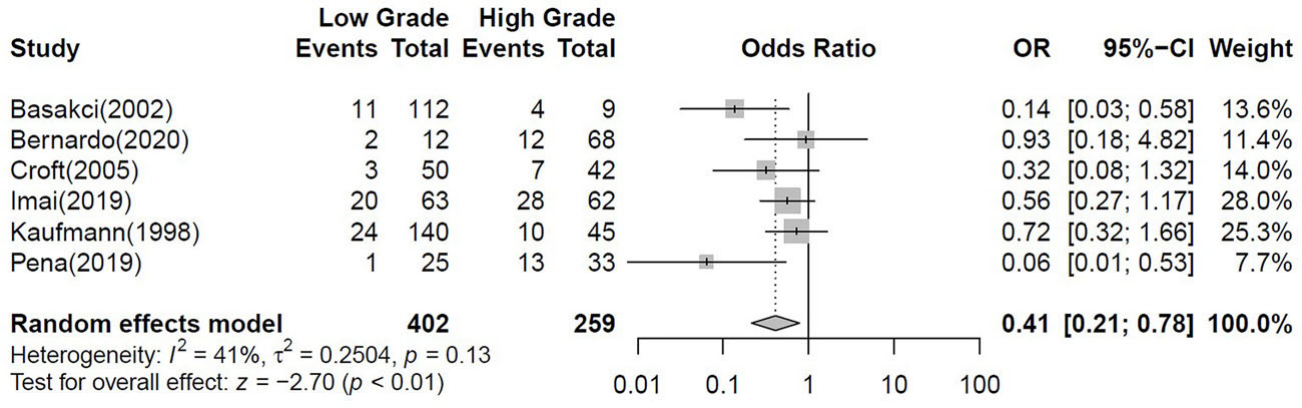
Forest plot for the binary meta-analysis stablishing the association between ERα positivity and tumour grade. Individual study estimates of crude odds ratios (OR) and 95% confidence intervals (CI). The diamond at the bottom of the plot denotes the random effects estimate. Error bars indicate confidence intervals. Heterogeneity was assessed using I2.

We conducted a binary meta-analysis to establish the correlation of ERα-positive cases with clinicopathological parameters: gender, age, tumour grade, tumour stage and histology (**Table 4**). Gender analysis (n=849 pooled cases from 7 studies; I2 = 0%) disclosed no significant differences between males and females CI= [0.43; 1.02], however, there was a tendency for a lower ERα expression in males (p=0.06). There was no difference in the age at diagnosis (n=230 from 3 studies; I2 = 0%) between ERα-positive and negative cases. ERα expression was significantly higher in high grade tumours (n=661 from 6 studies; I^2^ = 41%, CI= [0.21-0.78], p-value< 0.01; **Figure 3**). For stage analysis, data from 4 studies (I^2^ = 5%) was divided as Ta+T1 (218 cases) and >=T2 (136 cases) and no significant association was found (CI= [0.31-1.04], although there was tendency for higher ERα-positivity in late-stage tumours. The association with histology could only be inferred from a single study (n=125) with low number of VH cases and showed that the proportion of ERα-positive cases was lower in UC tumours when compared with either VH or UCDD (**Table 4**).

**Table 4:** Meta-analysis summary table.

Stratification	Protein	No. Of Studies	Patients (n)	Pooled OR (95% CI)		Heterogeneity	
				Random	p value	I2 (%)	p value
**Gender**	ERα	7	849	0.66 [0.43; 1.02]	0.06	0	0.92
	ERβ	2	268	1.80 [0.91; 3.57]	0.09	0	0.79
	GATA3	10	961	1.53 [1.02; 2.29]*	0.04	0	0.73
**Tumour Stage**	ERα	4	354	0.57 [0.31; 1.04]	0.07	5	0.37
	ERβ	4	583	0.77 [0.18; 3.33]	0.72	91	<0.01
	GATA3	7	1040	4.73 [2.18; 10.28]*	< 0.01	38	0.14
**Lymph node metastases**	ERα						
	ERβ	2	309	2.62 [1.25; 5.48]*	0.01	0	0.40
	GATA3	5	453	0.88 [0.37; 2.10]	0.78	54	0.07
**Tumour Grade**	ERα	6	661	0.41 [0.21; 0.78]*	< 0.01	41	0.13
	ERβ	5	657	1.08 [0.34; 3.49]	0.89	86	<0.01
	GATA3	9	1253	4.14 [1.79; 9.54]*	< 0.01	38	0.11
**Histology UC *vs* VH**	ERα	1	105	2.55 [0.41; 16]	0.32	NA	NA
	ERβ						
	GATA3	9	991	0.08 [0.03; 0.18]*	<0.01	52	0.03
**Histology UC *vs* UCDD**	ERα	1	120	1.14 [0.43; 3.03]	0.80	NA	NA
	ERβ						
	GATA3	10	758	0.21 [0.08; 0.53]*	<0.01	50	0.03
**Histology UCDD *vs* VH**	ERα	1	25	0.44 [0.06; 3.29]	0.43	NA	NA
	ERβ						
	GATA3	8	354	2.55 [0.45; 14.66]	0.29	82	<0.01
**Therapy pre-collection**	ERα						
	ERβ	1	72	1.12 [0.44; 2.83]	0.81	NA	NA
	GATA3						
				**Pooled MD (95% CI)**			
				**Random**	**p value**		
**Age**	ERα	3	230	0.77 [-3.08; 4.62]	0.69	0	0.97
	ERβ	2	268	-2.22 [-5.64; 1.20]	0.20	0	0.43
	GATA3	5	283	7.41 [1.90; 12.92]*	<0.01	66	0.02

UC, urothelial carcinoma; UCDD, urothelial carcinoma with divergent differentiation; VH, variant histology; NA, not applicable. *significant association.

### Meta-Analysis of ERβ Expression in BlaCa

Four hundred and thirty samples pooled from 5 studies were ERβ-positive (**Figure S5**), corresponding to 69% of the cases (range: 49–91%; I2 = 94%). Neither subgroup analysis nor meta-regression could explain the source of heterogeneity (**Tables S2** and **S3**) and a sensitivity analysis showed that selective omission of each study did not influence the overall heterogeneity (**Figure S4B**).

#### Correlation of ERβ Immunostaining With Clinicopathological Parameters

**Figure 4:**
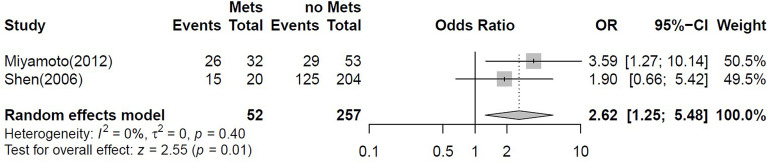
Forest plot for the binary meta-analysis stablishing the association between ERβ positivity and lymph node metastasis. Individual study estimates of crude odds ratios (OR) and 95% confidence intervals (CI). The diamond at the bottom of the plot denotes the random effects estimate. Error bars indicate confidence intervals. Heterogeneity (I2).

A binary meta-analysis was conducted to evaluate the association of ERβ positivity with patients’ gender, tumour stage, grade, presence of lymph node metastasis and patients’ pre-operative treatment (**Table 4**). Variation between studies was high and no significant association was found between ERβ expression and the clinicopathological parameters evaluated except for lymph node metastases. ERβ-positive cases were significantly correlated with the presence of lymph node metastasis [n=309 from 2 studies (32, 44)] (**Figure 4**).

### Meta-Analysis of GATA3 Expression in BlaCa

GATA3 was expressed in 85% of the 4275 pooled cases from 58 studies (range: 3-100%; **Figure S6**). Despite the high level of heterogeneity (I2 = 97%), the sensitivity analysis did not identify any study as having a large influence in the overall results (**Figure S4C**). However, data from Liang (2014) stands out and influences pooled results possibly due to the higher number of UCDD cases analysed (79). However, this trend did not reach significance in the subgroup meta-analysis (**Tables S2**, **S3**) indicating that technical variations or cohort composition were not the main drivers of heterogeneity. Meta regression was used to estimate whether the heterogeneity between studies was explained by clinicopathological covariates (**Table S3**). Interestingly, tumour stage, grade and pre-operative therapy significantly affected GATA3 positivity (p-value for mixed-effects model, p=0.0409, p=0.0056, p=0.0006, respectively).

#### Correlation of GATA3 Immunostaining With Clinicopathological Parameters

**Figure 5:**
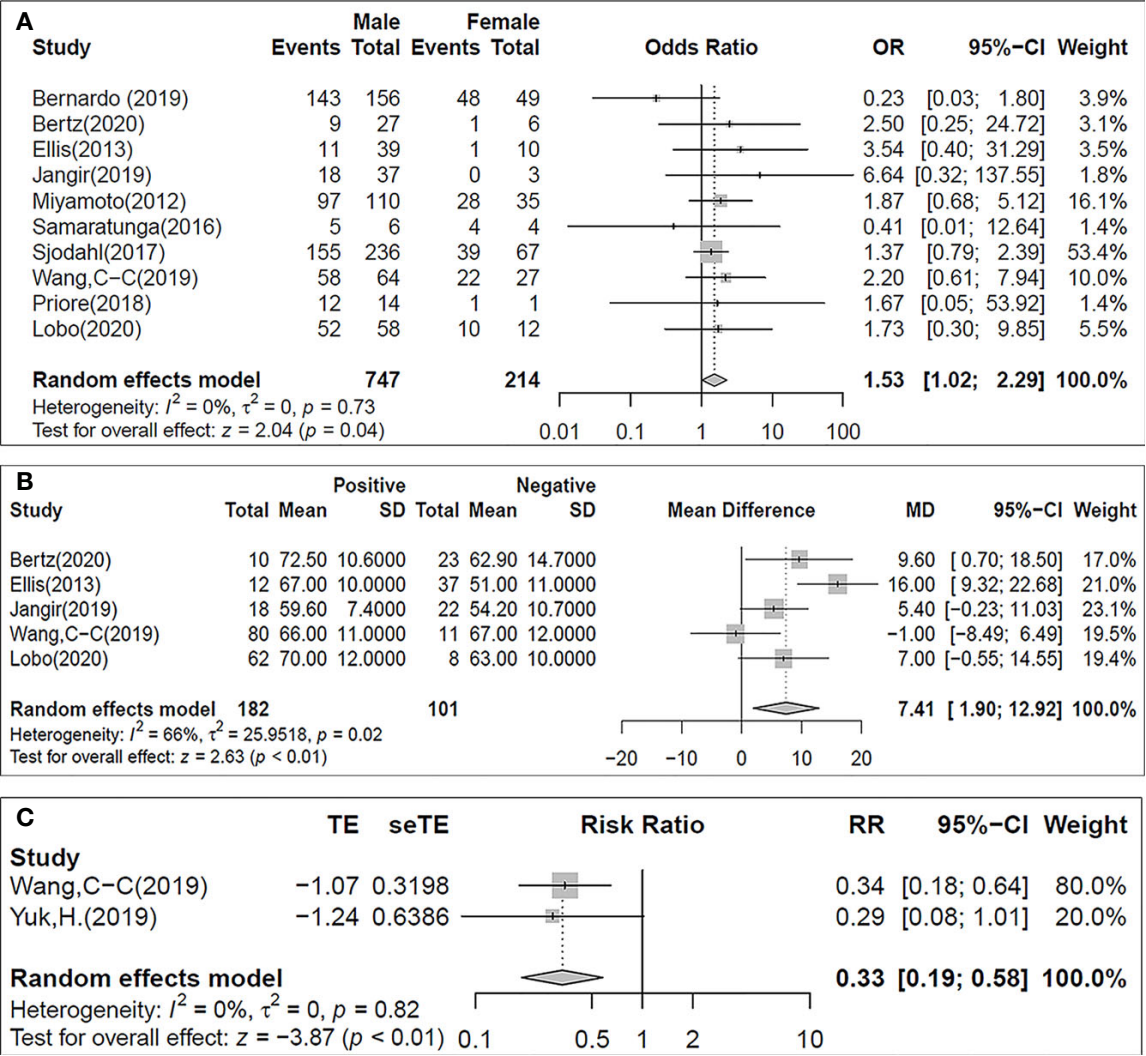
Forest plots for the binary meta-analysis stablishing the association between GATA3 positivity and the patients’ sex **(A)**, age at the time of surgery **(B)** and the recurrence free survival [RFS; **(C)**]. Individual study estimates of crude odds ratios (OR) and 95% confidence intervals (CI). The diamond at the bottom of the plot denotes the random effects estimate. Error bars indicate confidence intervals. Heterogeneity (I2).

The association of GATA3 positivity with patients’ gender, tumour stage, grade, histology and the presence of lymph node metastasis was evaluated in a binary meta-analysis (**Table 4**). Gender analysis (n=961, from 10 studies; I2 = 0%) disclosed a significantly higher proportion of GATA3-positive cases in males (CI= [1.02; 2.29]; **Figure 5A**). There was significantly higher expression in tumours from older patients (n=282, from 5 studies; I2 = 66%, CI= [1.90; 12.92]; **Figure 5B**). Two studies were included in the meta-analysis of GATA3 expression and recurrence free survival (RFS), analysing a total of 172 positive samples in a cohort of 192 patients. GATA3 expression was significantly associated with lower risk of recurrence (I2 = 0%; RR= 0.33; CI = [0.19; 0.58], p-value< 0.01; **Figure 5C**). Although this conclusion deserves to be followed up with a higher number of studies, the effect was strong and reflected the results of the individual studies included in this analysis (101, 103).

**Figure 6:**
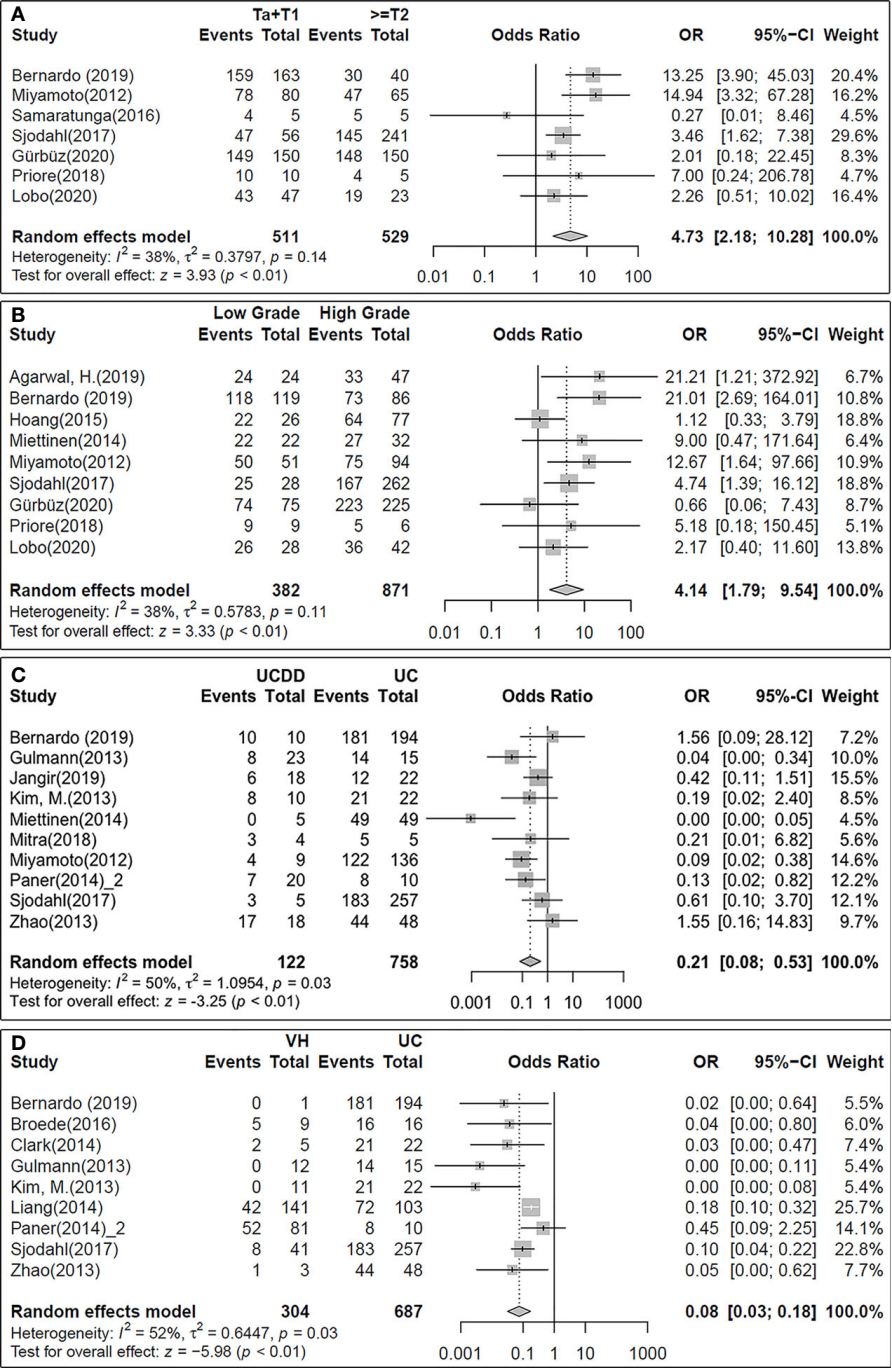
Forest plots for the binary meta-analysis showing the association between GATA3 positivity and the clinicopathological parameters tumour stage **(A)**, tumour grade **(B)** and histological differentiation of the tumours **(C, D)**.

GATA3 expression was found significantly higher in low stage (Ta+T1) compared with invasive tumours (>=T2) (CI= [2.18; 10.28], p-value< 0.01; **Figure 6A**) in the stage analysis (n=1040, from 7 studies; I2 = 38%). However, no significant correlation was found between GATA3 expression and lymph node metastasis. Similarly, GATA3 expression was significantly higher in low grade tumours as shown in the tumour grade analysis (n=1253, from 9 studies; I2 = 38%, CI= [1.79; 9.54], p-value< 0.01; **Figure 6B**). Tumour histology analysis revealed significantly higher GATA3 positivity in UC when compared to UCDD (n=880, from 10 studies; I2 = 50%, CI = [0.08; 0.53], p-value< 0.01; **Figure 6C**) or VH tumours (n=991, from 9 studies; I2 = 52%, CI = [0.03; 0.18], p-value< 0.01) (**Figure 6D**). No difference was found between UCDD and VH tumours.

#### Association Between ERα, ERβ and GATA3

Network meta-analysis was performed to assess a possible relationship between the expression of ERα, ERβ and GATA3 (**Table 5**). The model was based on direct evidence pooled from studies evaluating at least two of the proteins in the same study: 3 studies for ERα and GATA3 (35, 45, 46), 2 studies for ERα and ERβ (27, 32) and 1 study for ERβ and GATA3 (86). The model showed that both ERβ (0.014; 95%; CI: 0.007-0.030) and GATA3 (0.002; 95%CI: 0.001- 0.005) positive cases negatively correlate with ERα-positivity. GATA3 positivity was also negatively associated with ERβ positive cases (0.168; 95%; CI: 0.098 - 0.290), even though the association wasn’t as strong as for ERα. Still, this associations should be interpreted with extreme caution as even though the studies evaluating ERβ used antibodies that to date were not found to be unspecific, there is still great controversy as to how to best detect ERβ by IHC and the number of studies is low. No disagreement/inconsistency between direct and indirect comparison were detected as significant (p = 0.936).

**Table 5:** Network meta-analysis summary table.

OR (95%CI)	ERα	ERβ	GATA3
**ERα**		0.014 (0.007;0.028)*	0.002 (0.001; 0.005)*
**ERβ**	Consistency (0.936)		0.168 (0.098; 0.291)*
**GATA3**	Consistency (0.936)	Consistency (0.936)	

*Significant association.

## Discussion

BlaCa is a heterogeneous disease for which to date, limited histopathological markers and therapeutic options exist. Gene expression signatures with GATA3 and active ER signalling characterize luminal BlaCa (15, 17) and disclose some similarities between luminal BlaCa and BC (107). In the breast, GATA3 is a necessary transcriptional coactivator of ERα-mediated proliferation (25, 108), both proteins cooperate to maintain the epithelial lineage and are diagnostic tools for luminal BC (109). However, it is unclear whether these proteins collaborate or have a role in luminal BlaCa pathophysiology. Since ERα is the gold standard for indication of hormonal therapy and both ERs can be targeted with hormonal therapy (110), disclosing the relationship between ERs and GATA3 is a necessary step to advance BlaCa diagnostics and therapeutics. To date, this is the first systematic review and meta-analysis addressing a potential relationship between ERs and GATA3 in BlaCa. Moreover, based on recent findings that disclosed a vast amount of anti-ERβ antibodies as unspecific (29, 30), we restricted the inclusion criteria to only include validated anti-ERβ antibodies.

To improve our understanding ERα, ERβ and GATA3 roles in BlaCa pathophysiology we defined the following questions *a priori*: 1) What is their prognostic value? 2) What is their diagnostic value? 3) How do clinicopathological parameters impact their expression? 4) What are the sources of heterogeneity and which can be controlled in future studies? and 5) Is expression of these three markers associated in any way?

### Prognostic Value

Six studies analysed the association between ERα and patients’ prognosis, with no significant association between ERα expression and tumour recurrence/progression or survival observed in each individual study (27, 33, 34, 39, 40). An exception was Pena et al. that showed less likelihood for tumour recurrence in ERα -positive cases using unadjusted logistic regression (46). Due to differences in the methodology and information reported, it was not possible to carry out a meta-analysis. Therefore, current data is still insufficient to determine the prognostic value of ERα. However, higher ERα-positivity was observed in late-stage and high-grade tumours not only in the present meta-analysis but also in individual studies (33, 36–38), which support the hypothesis that ERα positivity may be a marker of poor prognosis. Our analysis disclosed an association between ERα expression and higher-grade tumours. Moreover, cell line studies showed that blocking ERα signalling with antiestrogens reduces cancer cell viability (27, 28), and a case study reported regression of metastatic transitional cell carcinoma in response to tamoxifen (111). Aromatase expression in the tumour parenchyma and stroma has been found significantly associated with more than a 2-fold risk of bladder cancer recurrence and may be associated with advanced tumour stage and poorer survival outcomes (112), while aromatase in the tumour stroma was significantly associated to adverse pathologic variables and poorer overall survival (113). On the other hand, the predictive value of ERβ is debatable, one study found ERβ-positivity to be associated with worse prognosis for low-grade tumours and lower CSS in high-stage tumours (114), while another study didn’t find any correlation between ERβ positivity and tumour recurrence (42). Additionally, Kauffman et al. found that higher ERβ levels were predictive of worse RF and OS following cystectomy (115). In the current meta-analysis data was insufficient to determine the prognostic value of ERβ due to differences in the methodology and data reported among individual studies. Regarding GATA3, pooled analysis indicated that positive expression was significantly associated with lower risk of recurrence, which is in agreement with the results of the individual studies included in this analysis (112, 113) and others that didn’t meet including criteria (73). This result independently confirms the prognostic value of GATA3 immunohistochemical determination in BlaCa.

### Diagnostic Value

Out of the 13 eligible studies for ERα, 8 analysed the association between ERα and tumour stage, grade, histological type and/or presence of lymph node metastasis. Two studies evaluated ERα expression in cohorts with multiple histologies and none found significant differences among the different histological types (37, 40). In the current meta-analysis, the association between ERα and tumour histology type was inferred from a single study (37) due to mathematical limitations. However, ERα-positivity was found to be higher in VH and UCDD histological types and less frequent in UC. From the 6 studies analysing ERα among different grade and stage, four found ERα expression to be significantly associated with high grade and muscle invasive tumours (33, 36–38) as in this meta-analysis. These results suggest that ERα-positivity is associated with more advanced tumours. No significant associations were found between ERα positivity and lymph node metastasis in individual studies (34, 40), and the available data was not suitable for meta-analysis. In the case of ERβ, previous studies based on evaluation of mRNA showed that ERβ expression was associated with tumours of the luminal subtype (107). None of the eligible studies provided data to allow the investigation ERβ expression among different histological types. The correlation between ERβ expression and tumour grade was inconsistent among the 5 eligible studies. Two studies found significant association between positive expression and tumour grade but in opposing directions. One noted higher expression in low grade tumours (44) an another in high grade tumours (43). A trend association between positive expression and high grade (32) and no relationship found in the remaining 2 (27, 42). Out of the 5 studies, 4 investigated the relationship between ERβ expression and tumour stage. ERβ expression was found significantly associated with high stage tumours in 2 of them (32, 44). In the same cohorts, ERβ-positivity was also associated with lymph node metastasis as also observed in this meta-analysis. The association between ERβ expression and other clinicopathological variables remains to be investigated.

GATA3 is an established marker of luminal papillary bladder tumours which are the least aggressive tumours and still retain some of the features of the urothelial differentiation (98). Similar to the results of this meta-analysis, four studies reported significant association between GATA3 expression and low grade (51, 59, 71, 86) and low stage (51, 59, 71, 86) tumours. GATA3 expression showed a significant association with tumour histology, with higher expression in UC as opposed to VH and UCDD, both in individual studies (57, 60, 70, 77, 79, 84, 86, 90) and in our meta-analysis. This is not surprising given its role in urothelial differentiation. Contradictorily, individual studies found significant correlation between increased GATA3-positivity and cases with lymph node metastasis (86) while in another data set it was associated with lymph node negative cases (104). Only one of these studies had data suitable for our pooled analysis (86) which found no significant correlation between GATA3-positive cases and lymph node metastases.

### Impact of Age and Sex

The relationship between age and sex and ERα (27, 33, 36–39,) and ERβ-positivity was investigated in seven and two independent studies, respectively, revealing no significant associations. The meta-analysis of the pooled data didn’t reach statistical significance but, suggest that both ERα and ERβ are more frequent in tumours from female patients as compared to males, and no differences were observed regarding age. Considering the higher estrogen levels in females, even after menopause, this observation is aligned with epidemiological data showing less frequent but more aggressive BlaCa in females (24). In the case of GATA3, 10 studies reported expression levels by age and sex (57, 65, 73, 81, 86, 93, 96, 98, 101), but no significant associations were reported. In the current meta-analysis, GATA3 expression was more frequent in tumours from older patients and, although not significant, there was a trend for higher GATA3 expression in males. These results are in line with a recent study that identified differences in BlaCa molecular subtypes based on sex, with tumours from females expressing higher levels of basal genes and more frequently from the basal/squamous subtype, while tumours from male patients expressed higher levels of luminal markers (116). A reduction of estrogen levels, as observed in menopause, causes urogenital side-effects (117, 118) and may participate in the carcinogenic process by promoting an inflammatory environment (119, 120). Therefore, it can be argued that antiestrogen therapy as used in BC treatment, which report similar urinary side-effects as menopausal and post-menopausal women would result in higher risk of developing BlaCa. We found a case study reporting a 65-year-old woman who developed non-muscle invasive low-grade papillary urothelial carcinoma grade 1, one and a half year after starting on endocrine therapy with aromatase inhibitors (121). However, this would not be related to ERα signalling directly initiating urothelial cell transformation, but to the inflammatory environment resulting from lower estrogen levels or higher androgen/estrogen ratio. Moreover, a large cohort prospective study found no overall associations of HRT use and oral contraceptive use with reduced risk of BlaCa (122).

### Limitations of the Meta-Analysis

We found several sources of heterogeneity common to evaluation of ERα, ERβ or GATA3, which may limit this meta-analysis but will certainly elucidate variables to consider in future research studies. These involve the inclusion of tumour samples from patients previously submitted to local or systemic therapy, which varied across different studies and most of the times it was not possible to stratify results by therapy. This might contribute to protein expression fluctuations in response to treatment. Another source of heterogeneity might be the publication bias related to lower number of non-statistically significant results, which can be explained by lack of reporting or less detailed description of results (123). Heterogeneity in stage and grade may be explained by inclusion of recurrent tumours which may have the same stage and grade of a firstly diagnosed tumour but distinct expression profile (124). Differences between primary and recurrences were not taken into consideration for analysis as this information is not available. The exclusion of cases with preoperative treatment and *carcinoma in situ* was also observed in some studies, which affect the stage and grade of the tumours under analysis. Similarly, the source of tissue contributes to variations as observed by a lower proportion of positive cases in TMA cohorts than in studies using whole-tissue sections. Regarding GATA3 and ERβ, differences in tumour histology explained part of the heterogeneity, with more GATA3 positive cases among UC and UCDD than in VH tumours. Furthermore, for ER detection, the antibodies are also an important source of variability as, even though they are validated for IHC and clinical use, come may detect more than one ER isoform. For ERα, the use of the clones 6F11 and SP1 provided higher dispersion in ERα positive cases, while the 1D5 gave more consistent results. These monoclonal antibodies recognize different epitopes, 6F11 was raised against the full length ERα, 1D5 recognizes the N-terminus, while SP1 antibody recognizes the C-terminus of human ERα. Others have shown that 6F11 and 1D5 antibodies only bind the full-length protein (66kDa) and SP1 could in principle also detect splice variants of smaller size (36 kDa, 46 kDa) (116). In the case of ERβ, all studies using non-specific antibodies were excluded, limiting the meta-analysis to the clone 14C8, which has been independently validated by different groups (29, 117), and the polyclonal MYEB which, to date, has not been probed unspecific. However, clone 14C8 detects ERβ isoforms 1 and 2 which, at least *in vitro*, have different biological effects (118) and may be differentially expressed. For GATA3, antibody usage does not seem to have much influence in the results obtained as evidenced by *Kandalaft et al* that used two different antibodies (119). Additional sources of heterogeneity that weren’t explored in this meta-analysis might also be at play such as different technologies to perform IHC, sensitivity to recognize positivity by different pathologists, among others. Finally, we were not able to include absolute positive/negative proportions, leaving some studies out of the pooled analysis for individual clinicopathological parameters.

### Correlation Between ER and GATA3 Positivity

The network meta-analysis model showed that there is a negative correlation between ERα or ERβ positivity with GATA3 expression, being the effect stronger for ERα. This agrees with *Miyamoto et al* (60) that showed a negative correlation between GATA3 and ERβ expression. Furthermore, both individual studies and our meta-analysis, propose that ERα and ERβ are markers of bad prognosis (33, 36–38, 60, 120), while GATA3 is associated to lower risk of recurrence and more differentiated tumours (60, 73, 98, 115, 121). Moreover, while GATA3 is higher in males, ERα appears to be higher in females. Therefore, in BlaCa, ERs and GATA3 do not appear to cooperate as observed in BC. Interestingly, ERα and ERβ expression were also negatively correlated. ERβ has been shown to counteract ERα activation, at least in some contexts (118, 122), so it is possible that lower ERβ contributes to an even more aggressive phenotype in ERα-positive BlaCa. Notably, this small subset of tumours may be eligible for hormonal treatment.

## Concluding Remarks

This systematic review confirmed that ERα is expressed in a small proportion of bladder tumours (3 – 13%) and is associated with higher tumour grade and stage independently of tumour histological type. Even if the % of positive cases is low, the possibility of benefiting these subgroup of worse prognosis patients with endocrine therapy should be further explored.

Our analysis and evidence from cell lines and aromatase expression points to a role of ERα in the progression of the disease. Functional studies are needed to identify if ERα is in fact a driver of proliferation in this subgroup of high-grade tumours and the relationship with aromatase expression in order to understand if these patients can benefit from antiestrogen therapy. No conclusion could be reached regarding ERβ even though it is a signature marker for luminal BlaCa and detected by IHC in 69% cases. On the other hand, GATA3 is expressed in about 80% cases and associated with low grade and low risk of recurrence. Therefore, while we were able to confirm the prognostic value of GATA3 using data from two studies, more studies correlating these biomarkers with time to event endpoints are needed to establish their prognostic value. Interestingly, this meta-analysis highlighted that ERα expression is dissociated from GATA3. In fact, higher positivity for each protein was identified in different groups of tumours with GATA3 positive expression associated with well differentiated tumours and ERα with loss of urothelial differentiation. Therefore, these two proteins do not collaborate to maintain epithelial luminal differentiation as observed in BC (109) and instead, they either participate in different stages of tumour progression or may be required for growth of different cancer cell types. This should be further confirmed in prospective studies considering both markers in advanced tumours and pre-resection treatment.

## Data Availability Statement 

The original contributions presented in the study are included in the article/**Supplementary Material**. Further inquiries can be directed to the corresponding author.

## Author Contributions 

CB: Conceptualized the work, carried out literature and statistical analysis for ERs and drafted the manuscript. ID: updated the literature search for ERs carried out the search for GATA3 and statistical analysis of all data and helped draft the manuscript. FLM: updated the literature search for ERs carried out the search for GATA3 and statistical analysis of all data and helped draft the manuscript. FA: discussed results and critically read the manuscript. VA: coordinated the meta-analysis and conceptualized the work. LS: discussed results and critically read the manuscript. LH: conceptualized the work, analysed the results and drafted the manuscript. All authors contributed to the article and approved the submitted version.

## Funding

This research was funded by iBiMED research unit UID/BIM/04501/2013 (LAH and CB) and UIDB/04501/2020 and UIDP/04501/2020 (LH); MEDISIS (CENTRO-01-0246-FEDER-000018) and pAGE (CENTRO-01-0145-FEDER-000003) supported by Comissão de Coordenação e Desenvolvimento Regional do Centro. CB, LM and ID also thank the Portuguese Science and Technology Foundation-FCT for their PhD scholarships SFRH/BD/80855/2011, SFRH/BD/117818/2016 and SFRH/BD/123821/2016, respectively. The Center for Research and Development in Mathematics and Applications (CIDMA) through the Portuguese Foundation for Science and Technology (FCT - Fundação para a Ciência e a Tecnologia), references UIDB/04106/2020 and UIDP/04106/2020.

## Conflict of Interest

The authors declare that the research was conducted in the absence of any commercial or financial relationships that could be construed as a potential conflict of interest.

## Publisher’s Note

All claims expressed in this article are solely those of the authors and do not necessarily represent those of their affiliated organizations, or those of the publisher, the editors and the reviewers. Any product that may be evaluated in this article, or claim that may be made by its manufacturer, is not guaranteed or endorsed by the publisher.

## Supplementary Material

Supplementary Figure 1 Risk of bias in individual studies. +, low risk;?, unclear risk; -, high risk.

Supplementary Figure 2 Funnel plots showing the asymmetry and publication bias for ERα, ERβ and GATA3.

Supplementary Figure 3 Forest plot showing pooled results for ERα positive expression in bladder cancer samples.

Supplementary Figure 4 Baujat plots comparing the weight of each study to the overall heterogeneity for ERα **(A)**, ERβ **(B)** and GATA3 **(C)**.

Supplementary Figure 5 Forest plot showing the pooled results for ERβ positive expression in bladder cancer samples.

Supplementary Figure 6 Forest plot showing the pooled results GATA3 positive expression in bladder cancer samples.

## Abbreviations

BlaCa, bladder cancer; ER, estrogen receptor; SCC, squamous cell carcinoma; UC, urothelial carcinoma; UCDD, urothelial carcinoma with divergent differentiation; VH, variant histologies; MIBC, muscle invasive bladder cancer; NMIBC, non-muscle invasive bladder cancer; FPKM, fragments per kilobase million; TUR, Transurethral resection; TMA, tissue microarray; CYS, cystectomy.

## References

[1] Koss LG . Bladder Cancer From a Perspective of 40 Years. J Cell Biochem Suppl (1992) 16I(S16I):23–9. doi: 10.1002/jcb.240501305 1305684

[2] Spruck CH Ohneseit PF Gonzalez-Zulueta M Esrig D Miyao N Tsai YC . Two Molecular Pathways to Transitional Cell Carcinoma of the Bladder. Cancer Res (1994) 54(3):784–8. 8306342

[3] Dinney CPN McConkey DJ Millikan RE Wu X Bar-Eli M Adam L . Focus on Bladder Cancer. Cancer Cell (2004) 6(2):111–6. doi: 10.1016/j.ccr.2004.08.002 15324694

[4] Castillo-Martin M Domingo-Domenech J Karni-Schmidt O Matos T Cordon-Cardo C . Molecular Pathways of Urothelial Development and Bladder Tumorigenesis. Urol Oncol Semin Orig Investig (2010) 28(4):401–8. doi: 10.1016/j.urolonc.2009.04.019 20610278

[5] Stein JP Skinner DG . Radical Cystectomy for Invasive Bladder Cancer: Long-Term Results of a Standard Procedure. World J Urol (2006) 24(3):296–304. doi: 10.1007/s00345-006-0061-7 16518661

[6] von der Maase H . Long-Term Survival Results of a Randomized Trial Comparing Gemcitabine Plus Cisplatin, With Methotrexate, Vinblastine, Doxorubicin, Plus Cisplatin in Patients With Bladder Cancer. J Clin Oncol (2005) 23(21):4602–8. doi: 10.1200/JCO.2005.07.757 16034041

[7] Sylvester RJ van der Meijden APMM Oosterlinck W Witjes JA Bouffioux C Denis L . Predicting Recurrence and Progression in Individual Patients With Stage Ta T1 Bladder Cancer Using EORTC Risk Tables: A Combined Analysis of 2596 Patients From Seven EORTC Trials. Eur Urol (2006) 49(3):466–5; discussion 475–7. doi: 10.1016/j.eururo.2005.12.031 16442208

[8] Kacew A Sweis RF . FGFR3 Alterations in the Era of Immunotherapy for Urothelial Bladder Cancer. Front Immunol (2020) 11:575258. doi: 10.3389/fimmu.2020.575258 33224141 PMC7674585

[9] Flaig TW Spiess PE Agarwal N Bangs R Boorjian SA Buyyounouski MK . Bladder Cancer, Version 3.2020, NCCN Clinical Practice Guidelines in Oncology. J Natl Compr Cancer Netw (2020) 18(3):329–54. doi: 10.6004/jnccn.2020.0011 32135513

[10] Babjuk M Böhle A Burger M Capoun O Cohen D Compérat EM . EAU Guidelines on Non–Muscle-Invasive Urothelial Carcinoma of the Bladder: Update 2016. Eur Urol (2017) 71(3):447–61. doi: 10.1016/j.eururo.2016.05.041 27324428

[11] Alfred Witjes J Lebret T Compérat EM Cowan NC De Santis M Bruins HM . Updated 2016 EAU Guidelines on Muscle-Invasive and Metastatic Bladder Cancer. Eur Urol (2017) 71(3):462–75. doi: 10.1016/j.eururo.2016.06.020 27375033

[12] Sylvester RJ . How Well Can You Actually Predict Which Non–Muscle-Invasive Bladder Cancer Patients Will Progress? Eur Urol (2011) 60(3):431–3. doi: 10.1016/j.eururo.2011.06.001 21680084

[13] Eble JN Sauter G Epstein JI Sesterhenn IA . World Health Organization Classification of Tumours. Pathology and Genetics of Tumours of the Urinary System and Male Genital Organs. 1st Edition. Lyon: IARC Press (2004).

[14] Hartge P Harvey EB Linehan WM Silverman DT Sullivan JW Hoover RN . Unexplained Excess Risk of Bladder Cancer in Men. JNCI J Natl Cancer Inst (1990) 82(20):1636–40. doi: 10.1093/jnci/82.20.1636 2213906

[15] Damrauer JS Hoadley KA Chism DD Fan C Tiganelli CJ Wobker SE . Intrinsic Subtypes of High-Grade Bladder Cancer Reflect the Hallmarks of Breast Cancer Biology. Proc Natl Acad Sci USA (2014) 111(8):3110–5. doi: 10.1073/pnas.1318376111 PMC3939870 24520177

[16] The Cancer Genome Atlas Research Network Weinstein JN Akbani R Broom BM Wang W Verhaak RGW . Comprehensive Molecular Characterization of Urothelial Bladder Carcinoma. Nature (2014) 507(7492):315–22. doi: 10.1038/nature12965 PMC3962515 24476821

[17] Choi W Porten S Kim S Willis D Plimack ER Hoffman-Censits J . Identification of Distinct Basal and Luminal Subtypes of Muscle-Invasive Bladder Cancer With Different Sensitivities to Frontline Chemotherapy. Cancer Cell (2014) 25(2):152–65. doi: 10.1016/j.ccr.2014.01.009 PMC4011497 24525232

[18] Warrick JI Walter V Yamashita H Chung E Shuman L Amponsa VO . FOXA1, GATA3 and PPARγ Cooperate to Drive Luminal Subtype in Bladder Cancer: A Molecular Analysis of Established Human Cell Lines. Sci Rep (2016) 6(1):38531. doi: 10.1038/srep38531 27924948 PMC5141480

[19] Ou Z Wang Y Chen J Tao L Zuo L Sahasrabudhe D . Estrogen Receptor β Promotes Bladder Cancer Growth and Invasion via Alteration of miR-92a/DAB2IP Signals. Exp Mol Med (2018) 50(11):1–11. doi: 10.1038/s12276-018-0155-5 PMC6243995 30459405

[20] Eriksson P Aine M Veerla S Liedberg F Sjödahl G Höglund M . Molecular Subtypes of Urothelial Carcinoma Are Defined by Specific Gene Regulatory Systems. BMC Med Genomics (2015) 8(1):25. doi: 10.1186/s12920-015-0101-5 26008846 PMC4446831

[21] Usary J Llaca V Karaca G Presswala S Karaca M He X . Mutation of GATA3 in Human Breast Tumors. Oncogene (2004) 23:7669–78. doi: 10.1038/sj.onc.1207966 15361840

[22] Mehra R Varambally S Ding L Shen R Sabel MS Ghosh D . Identification of GATA3 as a Breast Cancer Prognostic Marker by Global Gene Expression Meta-Analysis. Cancer Res (2005) 65(24):11259–64. doi: 10.1158/0008-5472.CAN-05-2495 16357129

[23] Arnold JM Choong DYH Thompson ER Waddell N Lindeman GJ Visvader JE . Frequent Somatic Mutations of GATA3 in Non-BRCA1/BRCA2 Familial Breast Tumors, But Not in BRCA1-, BRCA2- or Sporadic Breast Tumors. Breast Cancer Res Treat (2010) 119(2):491–6. doi: 10.1007/s10549-008-0269-x 19189213

[24] Dobruch J Daneshmand S Fisch M Lotan Y Noon AP Resnick MJ . Gender and Bladder Cancer: A Collaborative Review of Etiology, Biology, and Outcomes. Eur Urol (2016) 69(2):300–10. doi: 10.1016/j.eururo.2015.08.037 26346676

[25] Theodorou V Stark R Menon S Carroll JS . GATA3 Acts Upstream of FOXA1 in Mediating ESR1 Binding by Shaping Enhancer Accessibility. Genome Res (2013) 23(1):12–22. doi: 10.1101/gr.139469.112 23172872 PMC3530671

[26] Deroo BJ Korach KS . Review Series Estrogen Receptors and Human Disease. J Clin Invest (2006) 116(3):561–70. doi: 10.1172/JCI27987 PMC2373424 16511588

[27] Bernardo C Santos J Costa C Tavares A Amaro T Marques I . Estrogen Receptors in Urogenital Schistosomiasis and Bladder Cancer: Estrogen Receptor Alpha-Mediated Cell Proliferation. Urol Oncol Semin Orig Investig (2020) 38(9):738.e23–738.e35. doi: 10.1016/j.urolonc.2020.04.022 32507545

[28] Hoffman KL Lerner SP Smith CL . Raloxifene Inhibits Growth of RT4 Urothelial Carcinoma Cells via Estrogen Receptor-Dependent Induction of Apoptosis and Inhibition of Proliferation. Horm Cancer (2013) 4(1):24–35. doi: 10.1007/s12672-012-0123-9 22965848 PMC3541450

[29] Andersson S Sundberg M Pristovsek N Ibrahim A Jonsson P Katona B . Insufficient Antibody Validation Challenges Oestrogen Receptor Beta Research. Nat Commun (2017) 8:15840. doi: 10.1038/ncomms15840 28643774 PMC5501969

[30] Nelson AW Groen AJ Miller JL Warren AY Holmes KA Tarulli GA . Comprehensive Assessment of Estrogen Receptor Beta Antibodies in Cancer Cell Line Models and Tissue Reveals Critical Limitations in Reagent Specificity. Mol Cell Endocrinol (2017) 440:138–50. doi: 10.1016/j.mce.2016.11.016 PMC5228587 27889472

[31] Ide H Inoue S Miyamoto H . Histopathological and Prognostic Significance of the Expression of Sex Hormone Receptors in Bladder Cancer: A Meta-Analysis of Immunohistochemical Studies. PLoS One (2017) 12(3):e0174746. doi: 10.1371/journal.pone.0174746 28362839 PMC5375178

[32] Shen SS Smith CL Hsieh J-TT Yu J Kim IY Jian W . Expression of Estrogen Receptors-Alpha and -Beta in Bladder Cancer Cell Lines and Human Bladder Tumor Tissue. Cancer (2006) 106(12):2610–6. doi: 10.1002/cncr.21945 16700038

[33] Basakci A Kirkali Z Tuzel E Yorukoglu K Mungan MU Sade M . Prognostic Significance of Estrogen Receptor Expression in Superficial Transitional Cell Carcinoma of the Urinary Bladder. Eur Urol (2002) 41(3):342–5. doi: 10.1016/S0302-2838(02)00038-6 12180239

[34] Bolenz C Lotan Y Ashfaq R Shariat SFS . Estrogen and Progesterone Hormonal Receptor Expression in Urothelial Carcinoma of the Bladder. Eur Urol (2009) 56(6):1093–5. doi: 10.1016/j.eururo.2009.06.032 19596509

[35] Borhan WM Cimino-Mathews AM Montgomery EA Epstein JI . Immunohistochemical Differentiation of Plasmacytoid Urothelial Carcinoma From Secondary Carcinoma Involvement of the Bladder. Am J Surg Pathol (2017) 41(11):1570–5. doi: 10.1097/PAS.0000000000000922 28786878

[36] Croft PR Lathrop SL Feddersen RM Joste NE . Estrogen Receptor Expression in Papillary Urothelial Carcinoma of the Bladder and Ovarian Transitional Cell Carcinoma. Arch Pathol Lab Med (2005) 129(2):194–9. doi: 10.5858/2005-129-194-EREIPU 15679420

[37] Imai Y Noda S Matsuyama C Shimizu A Kamai T . Sex Steroid Hormone Receptors in Bladder Cancer: Usefulness in Differential Diagnosis and Implications in Histogenesis of Bladder Cancer. Urol Oncol Semin Orig Investig (2019) 37(6):353.e9–15. doi: 10.1016/j.urolonc.2019.01.023 30737158

[38] Kaufmann O Baume H Dietel M . Detection of Oestrogen Receptors in Non-Invasive and Invasive Transitional Cell Carcinomas of the Urinary Bladder Using Both Conventional Immunohistochemistry and the Tyramide Staining Amplification (TSA) Technique. J Pathol (1998) 186(2):165–8. doi: 10.1002/(SICI)1096-9896(1998100)186:2<165::AID-PATH155>3.0.CO;2-Y 9924432

[39] Mashhadi R Pourmand G Kosari F Mehrsai A Salem S Pourmand MR . Role of Steroid Hormone Receptors in Formation and Progression of Bladder. Urol J (2014) 11(6):1968–73. 25433476

[40] Tan W Boorjian S Advani P Farmer S Lohse C Cheville J . The Estrogen Pathway: Estrogen Receptor-α, Progesterone Receptor, and Estrogen Receptor-β Expression in Radical Cystectomy Urothelial Cell Carcinoma Specimens. Clin Genitourin Cancer (2015) 13(5):476–84. doi: 10.1016/j.clgc.2015.04.001 25981333

[41] Wei S Said-Al-Naief N Hameed O . Estrogen and Progesterone Receptor Expression Is Not Always Specific for Mammary and Gynecologic Carcinomas. Appl Immunohistochem Mol Morphol (2009) 17(5):393–402. doi: 10.1097/PAI.0b013e31819faa07 19417624

[42] Izumi K Ito Y Miyamoto H Miyoshi Y Ota J Moriyama M . Expression of Androgen Receptor in Non-Muscle-Invasive Bladder Cancer Predicts the Preventive Effect of Androgen Deprivation Therapy on Tumor Recurrence. Oncotarget (2016) 7(12):14153–60. doi: 10.18632/oncotarget.7358 PMC4924704 26885620

[43] Kontos S Papatsoris A Kominea A Melachrinou M Tanoglidi A Kachrilas S . Expression of Erβ and Its Co-Regulators P300 and NCoR in Human Transitional Cell Bladder Cancer. Urol Int (2011) 87(2):151–8. doi: 10.1159/000324262 21525722

[44] Miyamoto H Yao JLL Chaux A Zheng Y Hsu I Izumi K . Expression of Androgen and Oestrogen Receptors and Its Prognostic Significance in Urothelial Neoplasm of the Urinary Bladder. BJU Int (2012) 109(11):1716–26. doi: 10.1111/j.1464-410X.2011.10706.x 22221549

[45] Wang Y Lu S Amin A Wang L . Coexpress of GATA-3 and ER in Anorectal and Head and Neck Squamous Cell Carcinoma Mimicking Metastatic Breast Cancer. Appl Immunohistochem Mol Morphol (2020) 29(6):409–13. doi: 10.1097/PAI.0000000000000887 33264107

[46] Rodriguez Pena MDC Chaux A Eich M-L Tregnago AC Taheri D Borhan W . Immunohistochemical Assessment of Basal and Luminal Markers in Non-Muscle Invasive Urothelial Carcinoma of Bladder. Virchows Arch (2019) 475(3):349–56. doi: 10.1007/s00428-019-02618-5 31300876

[47] Balduzzi S Rücker G Schwarzer G . How to Perform a Meta-Analysis With R: A Practical Tutorial. Evid Based Ment Health (2019) 22(4):153–60. doi: 10.1136/ebmental-2019-300117 PMC10231495 31563865

[48] Rücker G Krahn U König J Efthimiou O Schwarzer G . netmeta: Network Meta-Analysis Using Frequentist Methods. R Package Version 1.3-0. (2021). Available at: https://CRAN.R-project.org/package=netmeta.

[49] Cohen J . Statistical Power Analysis for the Behavioral Scieences. 2 edition. New York: Lawrence Erlbaum Associates Inc (1988).

[50] Higgins JPT Thompson SG . Quantifying Heterogeneity in a Meta-Analysis. Stat Med (2002) 21(11):1539–58. doi: 10.1002/sim.1186 12111919

[51] Agarwal H Babu S Rana C Kumar M Singhai A Shankhwar S . Diagnostic Utility of GATA3 Immunohistochemical Expression in Urothelial Carcinoma. Indian J Pathol Microbiol (2019) 62(2):244. doi: 10.4103/IJPM.IJPM_228_18 30971548

[52] Aphivatanasiri C Li J Chan R Jamidi SK Tsang JY Poon IK . Combined SOX10 GATA3 Is Most Sensitive in Detecting Primary and Metastatic Breast Cancers: A Comparative Study of Breast Markers in Multiple Tumors. Breast Cancer Res Treat (2020) 184(1):11–21. doi: 10.1007/s10549-020-05818-9 32737715

[53] Barth I Schneider U Grimm T Karl A Horst D Gaisa NT . Progression of Urothelial Carcinoma In Situ of the Urinary Bladder: A Switch From Luminal to Basal Phenotype and Related Therapeutic Implications. Virchows Arch (2018) 472(5):749–58. doi: 10.1007/s00428-018-2354-9 PMC5978840 29654370

[54] Beltran AL Montironi R Cheng L . Microcystic Urothelial Carcinoma: Morphology, Immunohistochemistry and Clinical Behaviour. Histopathology (2014) 64(6):872–9. doi: 10.1111/his.12345 24321001

[55] Beltran AL Cheng L Montironi R Blanca A Leva M Rouprêt M . Clinicopathological Characteristics and Outcome of Nested Carcinoma of the Urinary Bladder. Virchows Arch (2014) 465(2):199–205. doi: 10.1007/s00428-014-1601-y 24878757

[56] Bernardo C Eriksson P Marzouka NA Liedberg F Sjödahl G Höglund M . Molecular Pathology of the Luminal Class of Urothelial Tumors. J Pathol (2019) 249(3):308–18. doi: 10.1002/path.5318 PMC6851980 31232464

[57] Bertz S Stöhr R Gaisa NT Wullich B Hartmann A Agaimy A . TERT Promoter Mutation Analysis as a Surrogate to Morphology and Immunohistochemistry in Problematic Spindle Cell Lesions of the Urinary Bladder. Histopathology (2020) 77(6):949–62. doi: 10.1111/his.14206 32645760

[58] Bezerra SM Lotan TL Faraj SF Karram S Sharma R Schoenberg M . GATA3 Expression in Small Cell Carcinoma of Bladder and Prostate and Its Potential Role in Determining Primary Tumor Origin. Hum Pathol (2014) 45(8):1682–7. doi: 10.1016/j.humpath.2014.04.011 24925221

[59] Bontoux C Rialland T Cussenot O Compérat E . A Four-Antibody Immunohistochemical Panel can Distinguish Clinico-Pathological Clusters of Urothelial Carcinoma and Reveals High Concordance Between Primary Tumor and Lymph Node Metastases. Virchows Arch (2020) 478(4):637–45. doi: 10.1007/s00428-020-02951-0 33128085

[60] Broede A Oll M Maurer A Siegert S Stoerkel S Golz R . Differential Diagnosis of Bladder Versus Colorectal Adenocarcinoma: Keratin 7 and GATA3 Positivity in Nuclear ß-Catenin-Negative Glandular Tumours Defines Adenocarcinoma of the Bladder. J Clin Pathol (2016) 69(4):307–12. doi: 10.1136/jclinpath-2015-203144 26463756

[61] Chang A Amin A Gabrielson E Illei P Roden RB Sharma R . Utility of GATA3 Immunohistochemistry in Differentiating Urothelial Carcinoma From Prostate Adenocarcinoma and Squamous Cell Carcinomas of the Uterine Cervix, Anus, and Lung. Am J Surg Pathol (2012) 36(10):1472–6. doi: 10.1097/PAS.0b013e318260cde7 PMC3444740 22982890

[62] Clark BZ Beriwal S Dabbs DJ Bhargava R . Semiquantitative GATA-3 Immunoreactivity in Breast, Bladder, Gynecologic Tract, and Other Cytokeratin 7-Positive Carcinomas. Am J Clin Pathol (2014) 142(1):64–71. doi: 10.1309/AJCP8H2VBDSCIOBF 24926087

[63] Compérat E McKenney JK Hartmann A Hes O Bertz S Varinot J . Large Nested Variant of Urothelial Carcinoma: A Clinicopathological Study of 36 Cases. Histopathology. (2017) 71(5):703–10. doi: 10.1111/his.13280 28805264

[64] Davis DG Siddiqui MT Oprea-Ilies G Stevens K Osunkoya AO Cohen C . GATA-3 and FOXA1 Expression Is Useful to Differentiate Breast Carcinoma From Other Carcinomas. Hum Pathol (2016) 47(1):26–31. doi: 10.1016/j.humpath.2015.09.015 26527523

[65] Ellis CL Chang AG Cimino-Mathews A Argani P Youssef RF Kapur P . GATA-3 Immunohistochemistry in the Differential Diagnosis of Adenocarcinoma of the Urinary Bladder. Am J Surg Pathol (2013) 37(11):1756–60. doi: 10.1097/PAS.0b013e31829cdba7 24061521

[66] Eckstein M Jung R Weigelt K Sikic D Stöhr R Geppert C . Piwi-like 1 and -2 protein expression levels are prognostic factors for muscle invasive urothelial bladder cancer patients. Sci Rep (2018) 8(1):17693. doi: 10.1038/s41598-018-35637-4 30523270 PMC6283838

[67] Fatima N Osunkoya AO . GATA3 expression in sarcomatoid urothelial carcinoma of the bladder. Hum Pathol (2014) 45(8):1625–9. doi: 10.1016/j.humpath.2014.03.015 24824028

[68] Guo CC Bondaruk J Yao H Wang Z Zhang L Lee S . Assessment of Luminal and Basal Phenotypes in Bladder Cancer. Sci Rep (2020) 10(1):9743. doi: 10.1038/s41598-020-66747-7 32546765 PMC7298008

[69] Gruver AM Amin MB Luthringer DJ Westfall D Arora K Farver CF . Selective Immunohistochemical Markers to Distinguish Between Metastatic High-Grade Urothelial Carcinoma and Primary Poorly Differentiated Invasive Squamous Cell Carcinoma of the Lung. Arch Pathol Lab Med (2012) 136(11):1339–46. doi: 10.5858/arpa.2011-0575-OA 23106579

[70] Gulmann C Paner GP Parakh RS Hansel DE Shen SS Ro JY . Immunohistochemical Profile to Distinguish Urothelial From Squamous Differentiation in Carcinomas of Urothelial Tract. Hum Pathol (2013) 44(2):164–72. doi: 10.1016/j.humpath.2012.05.018 22995333

[71] Gürbüz BÇ Topal CS Sobay R Alkurt G Zemheri IE . Molecular and Immunohistochemical Evaluation of BAP-1 Antibody in Bladder Cancer and Comparison With Luminal-Basal Subtyping. Pathol - Res Pract (2021) 217:153308. doi: 10.1016/j.prp.2020.153308 33341088

[72] Hoang LL Tacha D Bremer RE Haas TS Cheng L Uroplakin II . (UPII), GATA3, and p40 are Highly Sensitive Markers for the Differential Diagnosis of Invasive Urothelial Carcinoma. Appl Immunohistochem Mol Morphol (2015) 23(10):711–6. doi: 10.1097/PAI.0000000000000143 25611245

[73] Jangir H Nambirajan A Seth A Sahoo RK Dinda AK Nayak B . Prognostic Stratification of Muscle Invasive Urothelial Carcinomas Using Limited Immunohistochemical Panel of Gata3 and Cytokeratins 5/6, 14 and 20. Ann Diagn Pathol (2019) 43:151397. doi: 10.1016/j.anndiagpath.2019.08.001 31494492

[74] Johnson SM Khararjian A Legesse TB Khani F Robinson BD Epstein JI . Nested Variant of Urothelial Carcinoma Is a Luminal Bladder Tumor With Distinct Coexpression of the Basal Marker Cytokeratin 5/6. Am J Clin Pathol (2021) 155(4):588–96. doi: 10.1093/ajcp/aqaa160 33118597

[75] Kandalaft PL Simon RA Isacson C Gown AM . Comparative Sensitivities and Specificities of Antibodies to Breast Markers GCDFP-15, Mammaglobin A, and Different Clones of Antibodies to GATA-3: A Study of 338 Tumors Using Whole Sections. Appl Immunohistochem Mol Morphol (2016) 24(9):609–14. doi: 10.1097/PAI.0000000000000237 26447897

[76] Kim B Lee C Kim YA Moon KC . PD-L1 Expression in Muscle-Invasive Urinary Bladder Urothelial Carcinoma According to Basal/Squamous-Like Phenotype. Front Oncol (2020) 10:527385. doi: 10.3389/fonc.2020.527385 33365265 PMC7750632

[77] Kim M Ro JY Amin MB de Peralta-Venturina M Kwon GY Park YW . Urothelial Eddies in Papillary Urothelial Neoplasms: A Distinct Morphologic Pattern With Low Risk for Progression. Int J Clin Exp Pathol (2013) 6(8):1458–66. PMC3726961 23923064

[78] Leivo MZ Elson PJ Tacha DE Delahunt B Hansel DE . A Combination of p40, GATA-3 and Uroplakin II Shows Utility in the Diagnosis and Prognosis of Muscle-Invasive Urothelial Carcinoma. Pathology (2016) 48(6):543–9. doi: 10.1016/j.pathol.2016.05.008 27594510

[79] Liang Y Heitzman J Kamat AM Dinney CP Czerniak B Guo CC . Differential Expression of GATA-3 in Urothelial Carcinoma Variants. Hum Pathol (2014) 45(7):1466–72. doi: 10.1016/j.humpath.2014.02.023 24745616

[80] Liu H Shi J Wilkerson ML Lin F . Immunohistochemical Evaluation of GATA3 Expression in Tumors and Normal Tissues: A Useful Immunomarker for Breast and Urothelial Carcinomas. Am J Clin Pathol (2012) 138(1):57–64. doi: 10.1309/AJCP5UAFMSA9ZQBZ 22706858

[81] Lobo J Monteiro-Reis S Guimarães-Teixeira C Lopes P Carneiro I Jerónimo C . Practicability of Clinical Application of Bladder Cancer Molecular Classification and Additional Value of Epithelial-to-Mesenchymal Transition: Prognostic Value of Vimentin Expression. J Transl Med (2020) 18(1):303. doi: 10.1186/s12967-020-02475-w 32758253 PMC7405371

[82] Lu J Zhang Y Wu C Chu C Liu Z Cao Y . Impact of Immunohistochemistry-Based Molecular Subtype on Predicting Chemotherapy Response and Survival in Patients With T1 Stage Bladder Cancer After Bladder-Preserving Treatment. Jpn J Clin Oncol (2021) 51(3):424–33. doi: 10.1093/jjco/hyaa219 33319245

[83] Manach Q Cussenot O Rouprêt M Gamé X Chartier-Kastler E Reus C . Analysis of Bladder Cancer Subtypes in Neurogenic Bladder Tumors. Can J Urol (2018) 25(1):9161–7. 29524970

[84] Miettinen M McCue PA Sarlomo-Rikala M Rys J Czapiewski P Wazny K . GATA3: A Multispecific But Potentially Useful Marker in Surgical Pathology: A Systematic Analysis of 2500 Epithelial and Nonepithelial Tumors. Am J Surg Pathol (2014) 38(1):13–22. doi: 10.1097/PAS.0b013e3182a0218f 24145643 PMC3991431

[85] Mitra S Chatterjee D Das A Gupta K Radotra BD Mandal AK . Urothelial Tumors With Villous Morphology: Histomorphology and Role of Immunohistochemistry in Diagnosis. APMIS. (2018) 126(3):191–200. doi: 10.1111/apm.12799 29399882

[86] Miyamoto H Izumi K Yao JL Li Y Yang Q McMahon LA . GATA Binding Protein 3 Is Down-Regulated in Bladder Cancer Yet Strong Expression Is an Independent Predictor of Poor Prognosis in Invasive Tumor. Hum Pathol (2012) 43(11):2033–40. doi: 10.1016/j.humpath.2012.02.011 22607700

[87] Mohammed KH Siddiqui MT Cohen C . GATA3 Immunohistochemical Expression in Invasive Urothelial Carcinoma. Urol Oncol (2016) 34(10):432.e9–432.e13. doi: 10.1016/j.urolonc.2016.04.016 27241168

[88] Mohanty SK Smith SC Chang E Luthringer DJ Gown AM Aron M . Evaluation of Contemporary Prostate and Urothelial Lineage Biomarkers in a Consecutive Cohort of Poorly Differentiated Bladder Neck Carcinomas. Am J Clin Pathol (2014) 142(2):173–83. doi: 10.1309/AJCPK1OV6IMNPFGL 25015857

[89] Paner GP Cox RM Richards K Akki A Gokden N Lopez-Beltran A . Pseudoangiosarcomatous Urothelial Carcinoma of the Urinary Bladder. Am J Surg Pathol (2014) 38(9):1251–9. doi: 10.1097/PAS.0000000000000241 25133708

[90] Paner GP Annaiah C Gulmann C Rao P Ro JY Hansel DE . Immunohistochemical Evaluation of Novel and Traditional Markers Associated With Urothelial Differentiation in a Spectrum of Variants of Urothelial Carcinoma of the Urinary Bladder. Hum Pathol (2014) 45(7):1473–82. doi: 10.1016/j.humpath.2014.02.024 24780825

[91] Patriarca C Comperat E Bollito E Ussia A Scola G Cavallero A . Whorled Urothelial Cell Carcinoma: A Neglected Variant. Int J Surg Pathol (2014) 22(5):408–13. doi: 10.1177/1066896914527608 24651908

[92] Perrino CM Ho A Dall CP Zynger DL . Utility of GATA3 in the Differential Diagnosis of Pheochromocytoma. Histopathology. (2017) 71(3):475–9. doi: 10.1111/his.13229 28374498

[93] Priore SF Schwartz LE Epstein JI . An Expanded Immunohistochemical Profile of Osteoclast-Rich Undifferentiated Carcinoma of the Urinary Tract. Mod Pathol (2018) 31(6):984–8. doi: 10.1038/s41379-018-0012-z 29410491

[94] Rao Q Williamson SR Lopez-Beltran A Montironi R Huang W Eble JN . Distinguishing Primary Adenocarcinoma of the Urinary Bladder From Secondary Involvement by Colorectal Adenocarcinoma: Extended Immunohistochemical Profiles Emphasizing Novel Markers. Mod Pathol (2013) 26(5):725–32. doi: 10.1038/modpathol.2012.229 23348900

[95] Raspollini MR Sardi I Giunti L Di Lollo S Baroni G Stomaci N . Plasmacytoid Urothelial Carcinoma of the Urinary Bladder: Clinicopathologic, Immunohistochemical, Ultrastructural, and Molecular Analysis of a Case Series. Hum Pathol (2011) 42(8):1149–58. doi: 10.1016/j.humpath.2010.11.011 21334719

[96] Samaratunga H Delahunt B Egevad L Adamson M Hussey D Malone G . Pleomorphic Giant Cell Carcinoma of the Urinary Bladder: An Extreme Form of Tumour De-Differentiation. Histopathology (2016) 68(4):533–40. doi: 10.1111/his.12785 26211928

[97] Sanfrancesco J McKenney JK Leivo MZ Gupta S Elson P Hansel DE . Sarcomatoid Urothelial Carcinoma of the Bladder: Analysis of 28 Cases With Emphasis on Clinicopathologic Features and Markers of Epithelial-to-Mesenchymal Transition. Arch Pathol Lab Med (2016) 140(6):543–51. doi: 10.5858/arpa.2015-0085-OA 27031776

[98] Sjödahl G Eriksson P Liedberg F Höglund M . Molecular Classification of Urothelial Carcinoma: Global mRNA Classification Versus Tumour-Cell Phenotype Classification. J Pathol (2017) 242(1):113–25. doi: 10.1002/path.4886 PMC5413843 28195647

[99] So JS Epstein JI . GATA3 Expression in Paragangliomas: A Pitfall Potentially Leading to Misdiagnosis of Urothelial Carcinoma. Mod Pathol (2013) 26(10):1365–70. doi: 10.1038/modpathol.2013.76 23599157

[100] Verduin L Mentrikoski MJ Heitz CT Wick MR . The Utility of GATA3 in the Diagnosis of Urothelial Carcinomas With Variant Morphologic Patterns. Appl Immunohistochem Mol Morphol (2016) 24(7):509–13. doi: 10.1097/PAI.0000000000000221 26317312

[101] Wang CC Tsai YC Jeng YM . Biological Significance of GATA3, Cytokeratin 20, Cytokeratin 5/6 and P53 Expression in Muscle-Invasive Bladder Cancer. PLoS One (2019) 14(8):e0221785. doi: 10.1371/journal.pone.0221785 31469885 PMC6716637

[102] Wang G Xiao L Zhang M Kamat AM Siefker-Radtke A Dinney CP . Small Cell Carcinoma of the Urinary Bladder: A Clinicopathological and Immunohistochemical Analysis of 81 Cases. Hum Pathol (2018) 79:57–65. doi: 10.1016/j.humpath.2018.05.005 29763719 PMC6133751

[103] Yuk HD Jeong CW Kwak C Kim HH Moon KC Ku JH . Clinical Outcomes of Muscle Invasive Bladder Cancer According to the BASQ Classification. BMC Cancer (2019) 19(1):897. doi: 10.1186/s12885-019-6042-1 31500577 PMC6734465

[104] Zhao L Antic T Witten D Paner GP Taxy JB Husain A . Is GATA3 Expression Maintained in Regional Metastases? Am J Surg Pathol (2013) 37(12):1876–81. doi: 10.1097/PAS.0b013e31829e2525 24121175

[105] Zinnall U Weyerer V Compérat E Camparo P Gaisa NT Knuechel-Clarke R . Micropapillary Urothelial Carcinoma: Evaluation of HER2 Status and Immunohistochemical Characterization of the Molecular Subtype. Hum Pathol (2018) 80:55–64. doi: 10.1016/j.humpath.2018.05.022 29885409

[106] McShane LM Altman DG Sauerbrei W Taube SE Gion M Clark GM . REporting Recommendations for Tumour MARKer Prognostic Studies (REMARK). Br J Cancer (2005) 93(4):387–91. doi: 10.1038/sj.bjc.6602678 PMC2361579 16106245

[107] Choi W Czerniak B Ochoa A Su X Siefker-Radtke A Dinney C . Intrinsic Basal and Luminal Subtypes of Muscle-Invasive Bladder Cancer. Nat Rev Urol (2014) 11(7):400–10. doi: 10.1038/nrurol.2014.129 24960601

[108] Eeckhoute J Keeton EK Lupien M Krum SA Carroll JS Brown M . Positive Cross-Regulatory Loop Ties GATA-3 to Estrogen Receptor α Expression in Breast Cancer. Cancer Res (2007) 67(13):6477–83. doi: 10.1158/0008-5472.CAN-07-0746 17616709

[109] Takaku M Grimm SA Wade PA . GATA3 in Breast Cancer: Tumor Suppressor or Oncogene? Gene Expr (2015) 16(4):163–8. doi: 10.3727/105221615X14399878166113 PMC4758516 26637396

[110] Lindberg K Helguero LA Omoto Y Gustafsson J-Å Haldosén L-A . Estrogen Receptor β Represses Akt Signaling in Breast Cancer Cells via Downregulation of HER2/HER3 and Upregulation of PTEN: Implications for Tamoxifen Sensitivity. Breast Cancer Res (2011) 13(2):R43. doi: 10.1186/bcr2865 21492444 PMC3219206

[111] Dellagrammaticas D Bryden AAG Collins GN . Regression of Metastatic Transitional Cell Carcinoma in Response to Tamoxifen. J Urol (2001) 165(5):1631. doi: 10.1097/00005392-200105000-00057 11342939

[112] Nguyen DP O’Malley P Al Hussein Al Awamlh B Furrer MA Mongan NP Robinson BD . Association of Aromatase With Bladder Cancer Stage and Long-Term Survival: New Insights Into the Hormonal Paradigm in Bladder Cancer. Clin Genitourin Cancer (2017) 15(2):256–62.e1. doi: 10.1016/j.clgc.2016.05.017 27324053

[113] Wu S Ye J Wang Z Lin SX Lu M Liang Y . Expression of Aromatase in Tumor Related Stroma Is Associated With Human Bladder Cancer Progression. Cancer Biol Ther (2018) 19(3):175–80. doi: 10.1080/15384047.2017.1414762 PMC5790358 29303414

[114] Miyamoto H Zheng Y Izumi K . Nuclear Hormone Receptor Signals as New Therapeutic Targets for Urothelial Carcinoma. Curr Cancer Drug Targets (2012) 12(1):14–22. doi: 10.2174/156800912798888965 22111835

[115] Kauffman EC Robinson BD Downes M Marcinkiewicz K Vourganti S Scherr DS . Estrogen Receptor-Beta Expression and Pharmacological Targeting in Bladder Cancer. Oncol Rep (2013) 30(1):131–8. doi: 10.3892/or.2013.2416 PMC3729232 23612777

[116] de Jong JJ Boormans JL van Rhijn BWG Seiler R Boorjian SA Konety B . Distribution of Molecular Subtypes in Muscle-Invasive Bladder Cancer Is Driven by Sex-Specific Differences. Eur Urol Oncol (2020) 3(4):420–3. doi: 10.1016/j.euo.2020.02.010 32205136

[117] Robinson D Cardozo LD . The Role of Estrogens in Female Lower Urinary Tract Dysfunction. Urology (2003) 62(4 Suppl 1):45–51. doi: 10.1016/S0090-4295(03)00676-9 14550837

[118] Hextall A . Oestrogens and Lower Urinary Tract Function. Maturitas (2000) 36(2):83–92. doi: 10.1016/S0378-5122(00)00143-2 11006496

[119] Kantor AF Hartge P Hoover RN Narayana AS Sullivan JW Fraumeni JF Jr . Urinary Tract Infection and Risk of Bladder Cancer. Am J Epidemiol (1984) 119(4):510–5. doi: 10.1093/oxfordjournals.aje.a113768 6711540

[120] Madeb R Messing EM . Gender, Racial and Age Differences in Bladder Cancer Incidence and Mortality. Urol Oncol Semin Orig Investig (2004) 22(2):86–92. doi: 10.1016/S1078-1439(03)00139-X 15082003

[121] Ntzeros K Stamatakos M Stokidis S Louka G . Bladder Cancer: The Hormonal Dependence Enigma and a New Hormonal Player. Indian J Surg (2015) 77(Suppl 3):1502–3. doi: 10.1007/s12262-014-1133-3 PMC4775549 27011619

[122] McGrath M Michaud DS De Vivo I . Hormonal and Reproductive Factors and the Risk of Bladder Cancer in Women. Am J Epidemiol (2006) 163(3):236–44. doi: 10.1093/aje/kwj028 16319290

[123] Kyzas PA Denaxa-Kyza D Ioannidis JPA . Almost All Articles on Cancer Prognostic Markers Report Statistically Significant Results. Eur J Cancer (2007) 43(17):2559–79. doi: 10.1016/j.ejca.2007.08.030 17981458

[124] Sjödahl G Eriksson P Patschan O Marzouka N Jakobsson L Bernardo C . Molecular Changes During Progression From Nonmuscle Invasive to Advanced Urothelial Carcinoma. Int J Cancer (2020) 146(9):2636–47. doi: 10.1002/ijc.32737 PMC7079000 31609466

