# Supplementary figures and images for: Association Between Estrogen Receptors and GATA3 in Bladder Cancer: A Systematic Review and Meta-Analysis of Their Clinicopathological Significance
Source: Front Endocrinol (Lausanne). 2021 Oct 8;12:684140. doi: 10.3389/fendo.2021.684140 (PMC8531553; doi:10.3389/fendo.2021.684140)

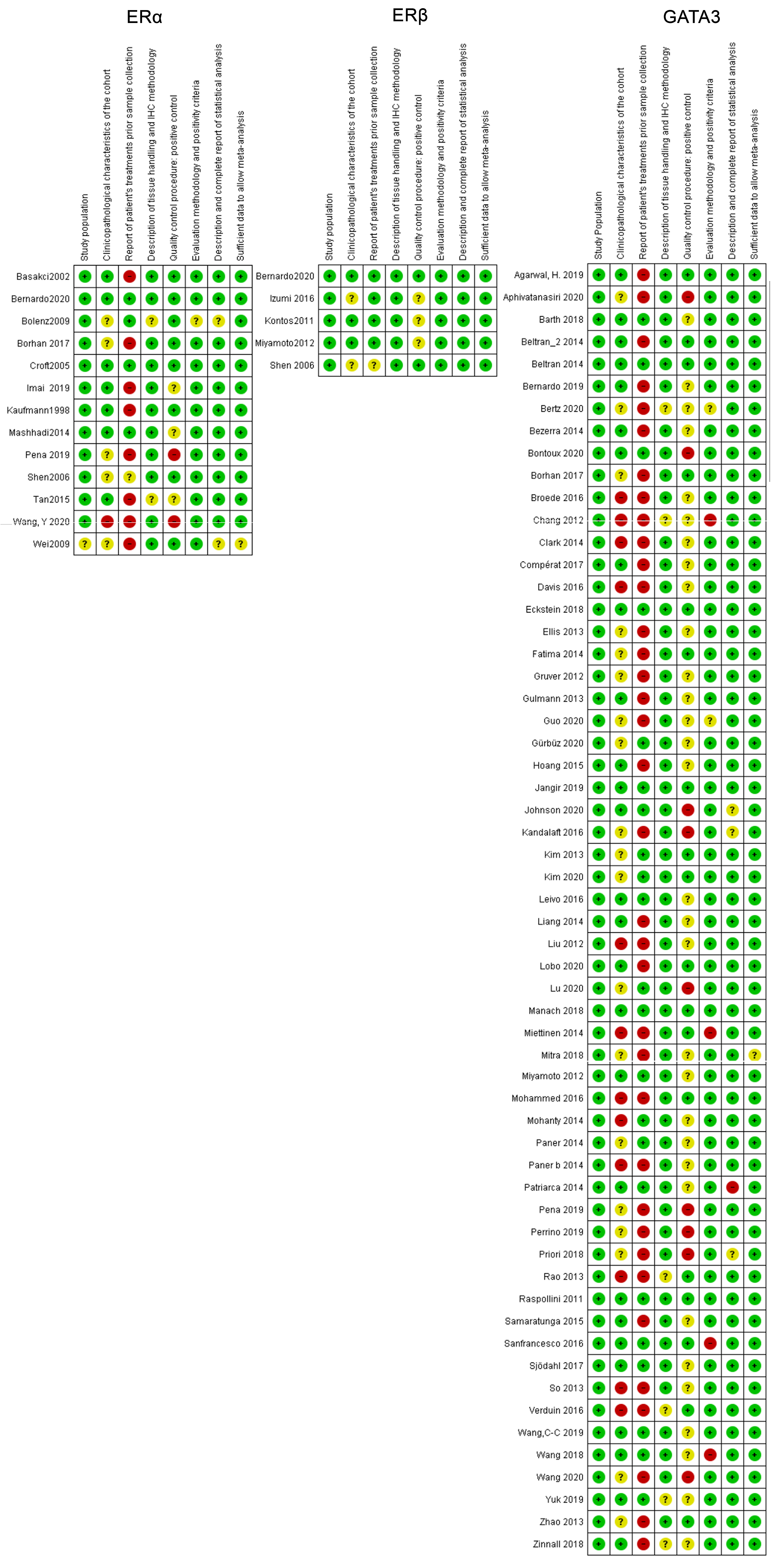

Supplement: Supplementary Figure 1 — Risk of bias in individual studies. +, low risk;?, unclear risk; -, high risk. [file Image_1.png]

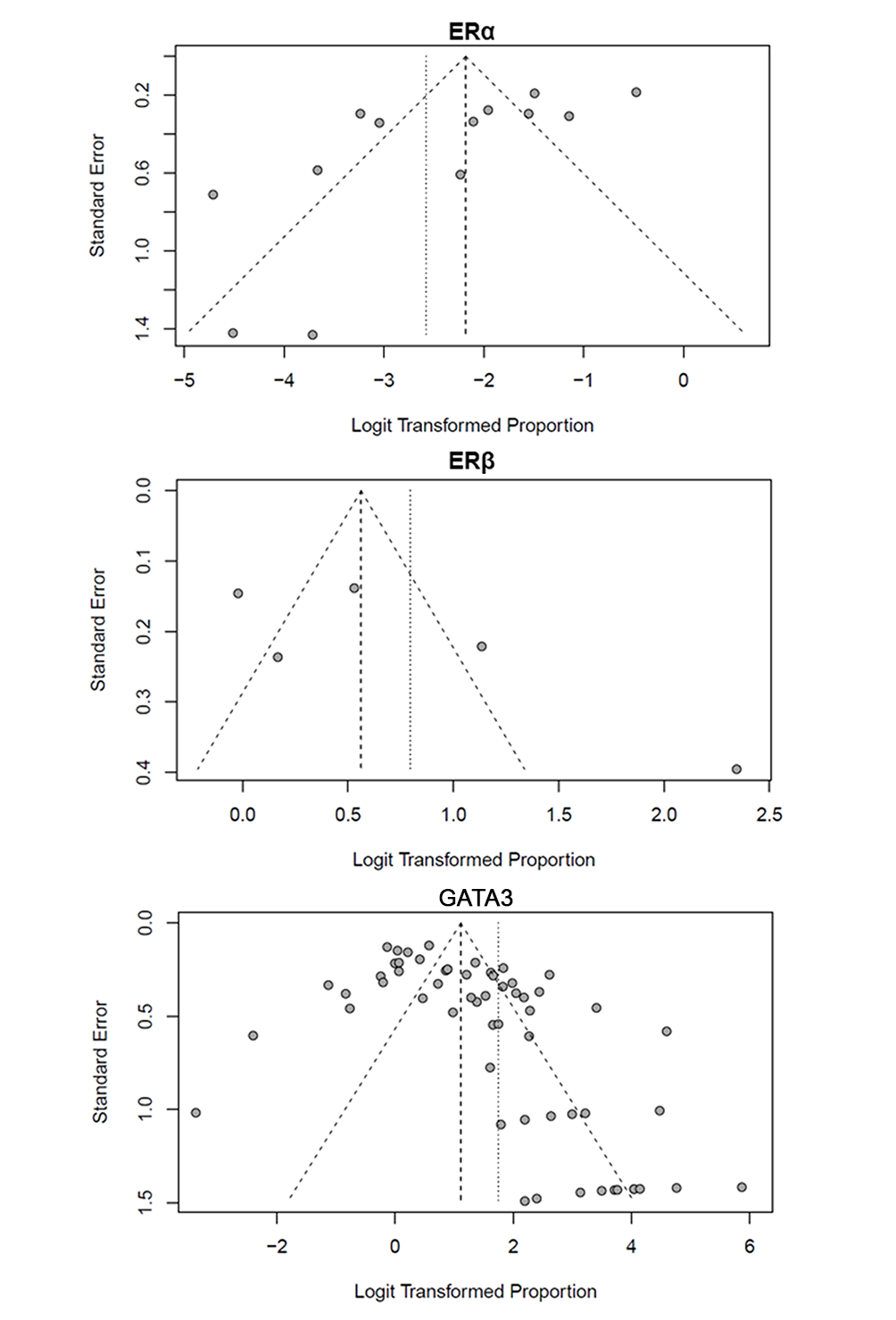

Supplement: Supplementary Figure 2 — Funnel plots showing the asymmetry and publication bias for ERα, ERβ and GATA3. [file Image_2.tif]

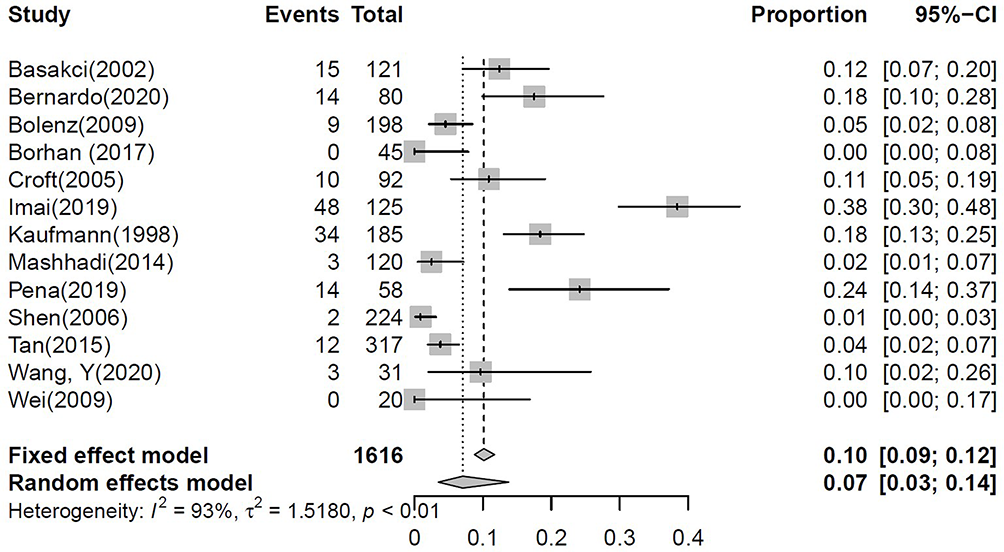

Supplement: Supplementary Figure 3 — Forest plot showing pooled results for ERα positive expression in bladder cancer samples. [file Image_3.tif]

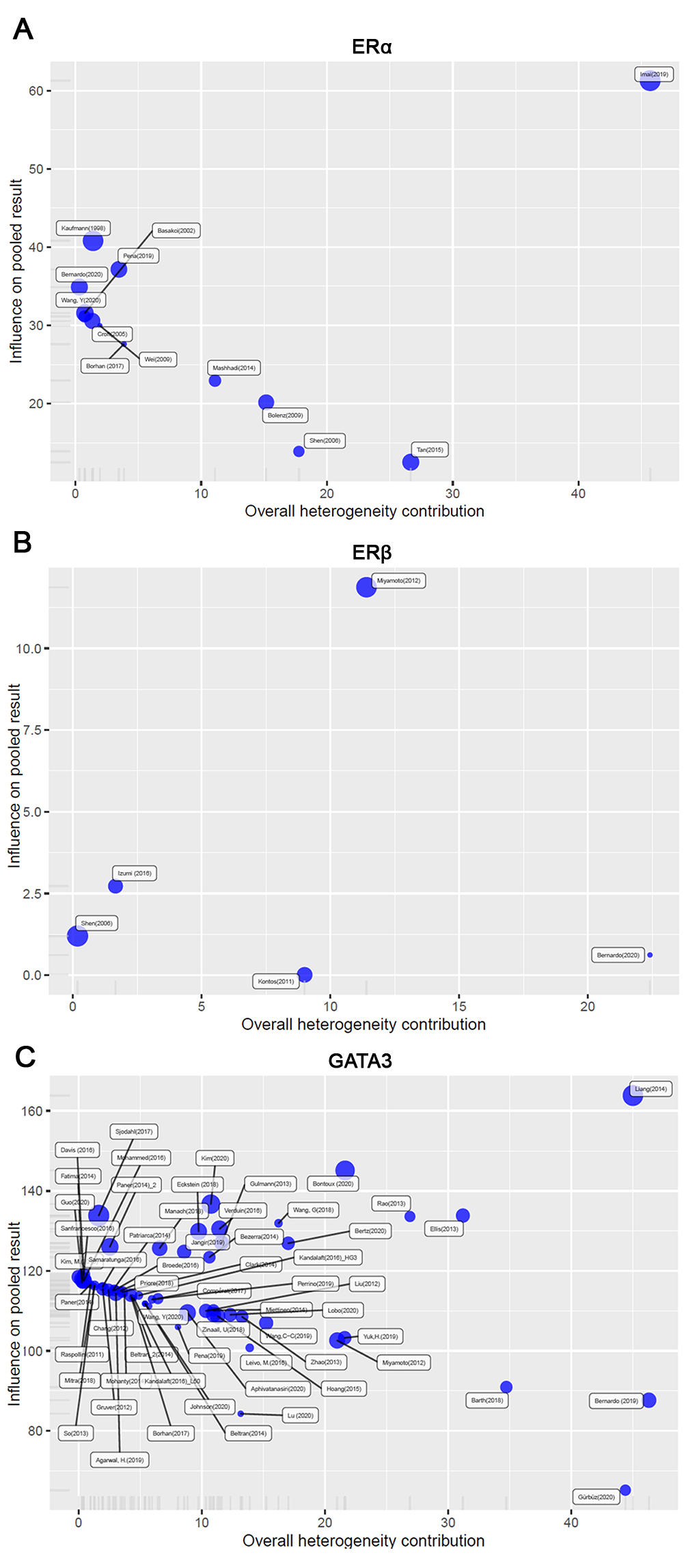

Supplement: Supplementary Figure 4 — Baujat plots comparing the weight of each study to the overall heterogeneity for ERα (A), ERβ (B) and GATA3 (C). [file Image_4.tif]

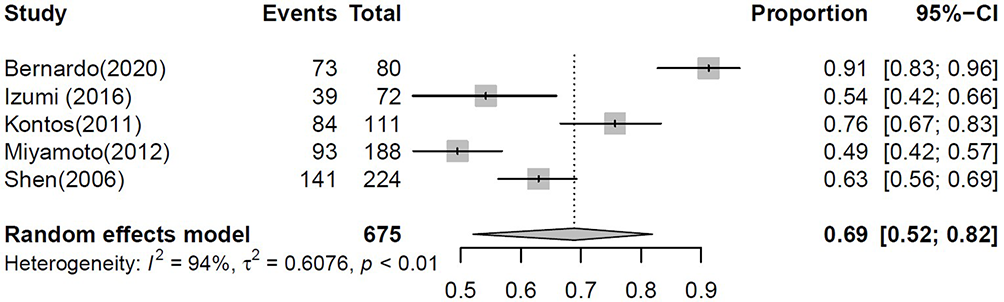

Supplement: Supplementary Figure 5 — Forest plot showing the pooled results for ERβ positive expression in bladder cancer samples. [file Image_5.tif]

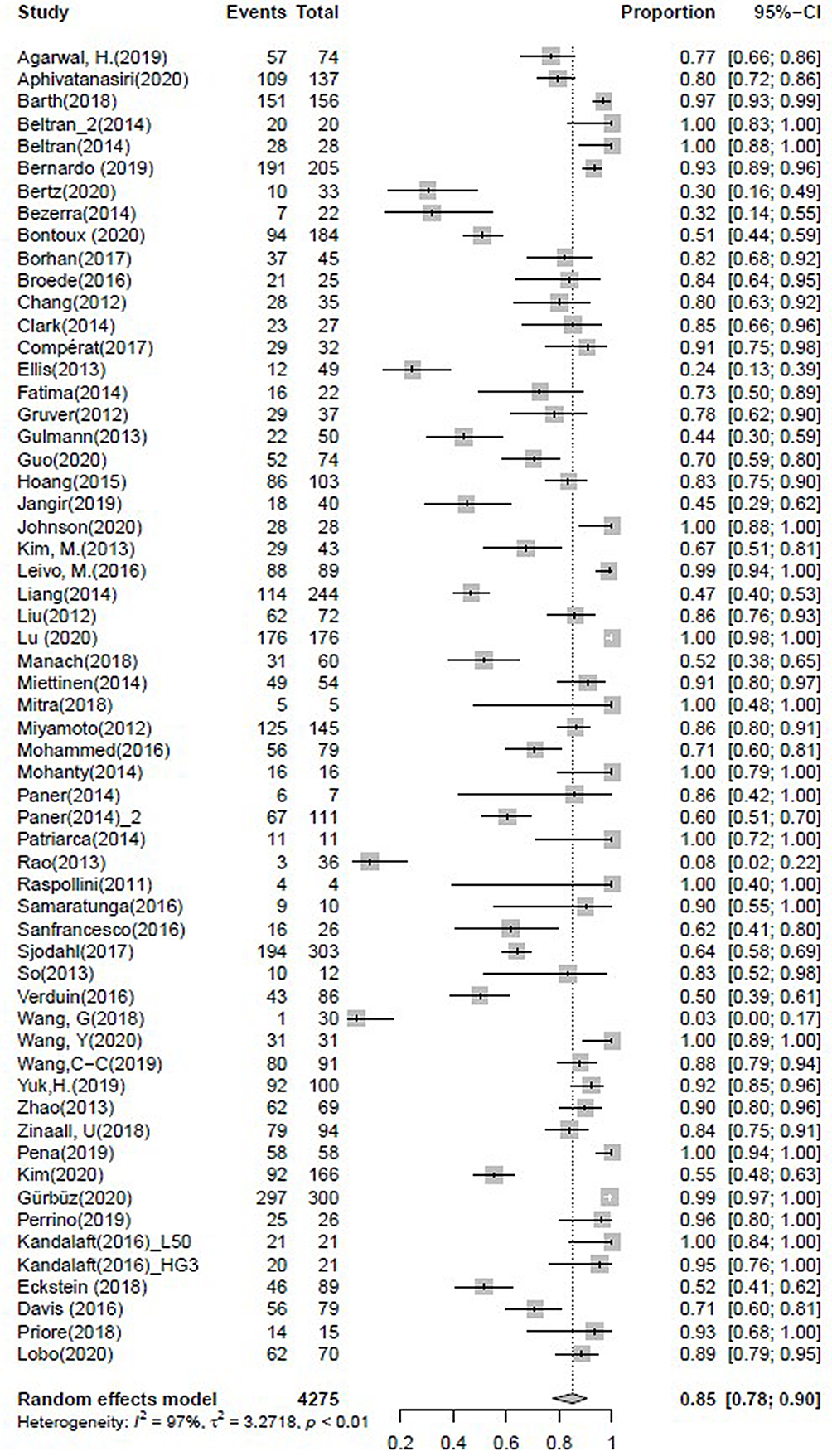

Supplement: Supplementary Figure 6 — Forest plot showing the pooled results GATA3 positive expression in bladder cancer samples. [file Image_6.tif]
